# Vegetation morphologic and aerodynamic characteristics reduce aeolian erosion

**DOI:** 10.1038/s41598-017-13084-x

**Published:** 2017-10-09

**Authors:** Abbas Miri, Deirdre Dragovich, Zhibao Dong

**Affiliations:** 1 Department of Watershed and Range Management, Faculty of Water and Soil, University of Zabol, Zabol, Iran; 2 School of Geosciences F09, University of Sydney, Sydney, NSW 2006 Australia; 3 Key Laboratory of Desert and Desertification, Cold and Arid Regions Environmental and Engineering Research Institute, Chinese Academy of Sciences, Lanzhou, 730000 Gansu China

## Abstract

Vegetation cover is crucial to controlling aeolian erosion but highly efficient vegetation is critical. How this efficiency is influenced by vegetation response to airflow is not clear. Here we evaluate the responses of *Cosmos bipinnatus* and *Ligustrum lucidum Ait* to a range of wind speeds in a wind tunnel. For both species, we calculate shelter effect and sand flux. We show that plant effectiveness in reducing wind speed and sediment transport is linked to their aerodynamic response to airflow which results from their morphology. We demonstrate that in low-density cover the flow-response and resistance of individuals is most critical in the optimal effectiveness of a canopy. Our wind tunnel experiment suggests that vegetation morphology and structure must be priority parameters in facilitating aeolian erosion control.

## Introduction

In the past few decades, aeolian erosion has become more significant because of changes in climate and vegetation cover. It has resulted in major environmental, climatic, economic and human-health problems in the form of dust storms in many areas of the world^1^. To prevent or reduce aeolian erosion, understanding the interaction between wind and the ground surface is important^2^. Vegetation plays a crucial role in reducing wind speed and thereby protecting the surface due to the complex internal and external geometry, porosity and flexibility^3^ of plants. Despite many advances in understanding the effectiveness of vegetation in controlling wind erosion^4–13^, studies have focused on the overall protective function of the vegetation, but our understanding of airflow processes and aeolian erosion in the presence of vegetation, and effects of vegetation characteristics, remains incomplete.

In several studies solid objects^11,14–17^, pieces of dead vegetation^18^ or artificial vegetation^19–23^ have been used to investigate the effect of roughness elements on airflow but the results from such studies may not be able to represent entirely the sheltering effect of live plants in natural environments. In addition, these studies have not experimentally considered plant aerodynamic responses to airflow in order to investigate the effect of plant morphology in aeolian erosion control. Little work has been done in wind tunnels to assess the reaction of live vegetation to airflow and blown sediment^10,12,23–26^. Thus, a more comprehensive and quantitative framework for selecting the type of vegetation required to minimise wind erosion in soil-erodible areas is still lacking. Identifying the effect of plant structure on sediment flux is a challenge in aeolian geomorphology^27–31^ but it is essential for scientific, modelling and aeolian research to understand how plant morphology contributes to more efficient erosion control through wind velocity reduction.

Here, we assess morphologic and aerodynamic responses of two types of live plants (one broad-leaved, the other narrow-leaved) to a range of wind speeds by conducting wind tunnel experiments. Although the particular plant species used in the current study are not suitable for reducing wind erosion in the field, the results obtained identify important vegetation characteristics which can be used as criteria for the selection of the most suitable plants from those that are available and most suitable in any region. Using live plants can significantly minimize experimental errors and allow for controlled within-experiment variations that are often not feasible under field conditions. Our goal is to understand how the aerodynamic and morphologic responses of different plant types in different densities impact their potential to affect wind velocity and thus aeolian sediment transport.

## Results and Discussion

### Morphologic and aerodynamic responses of two plant types to airflow

**Figure 1:**
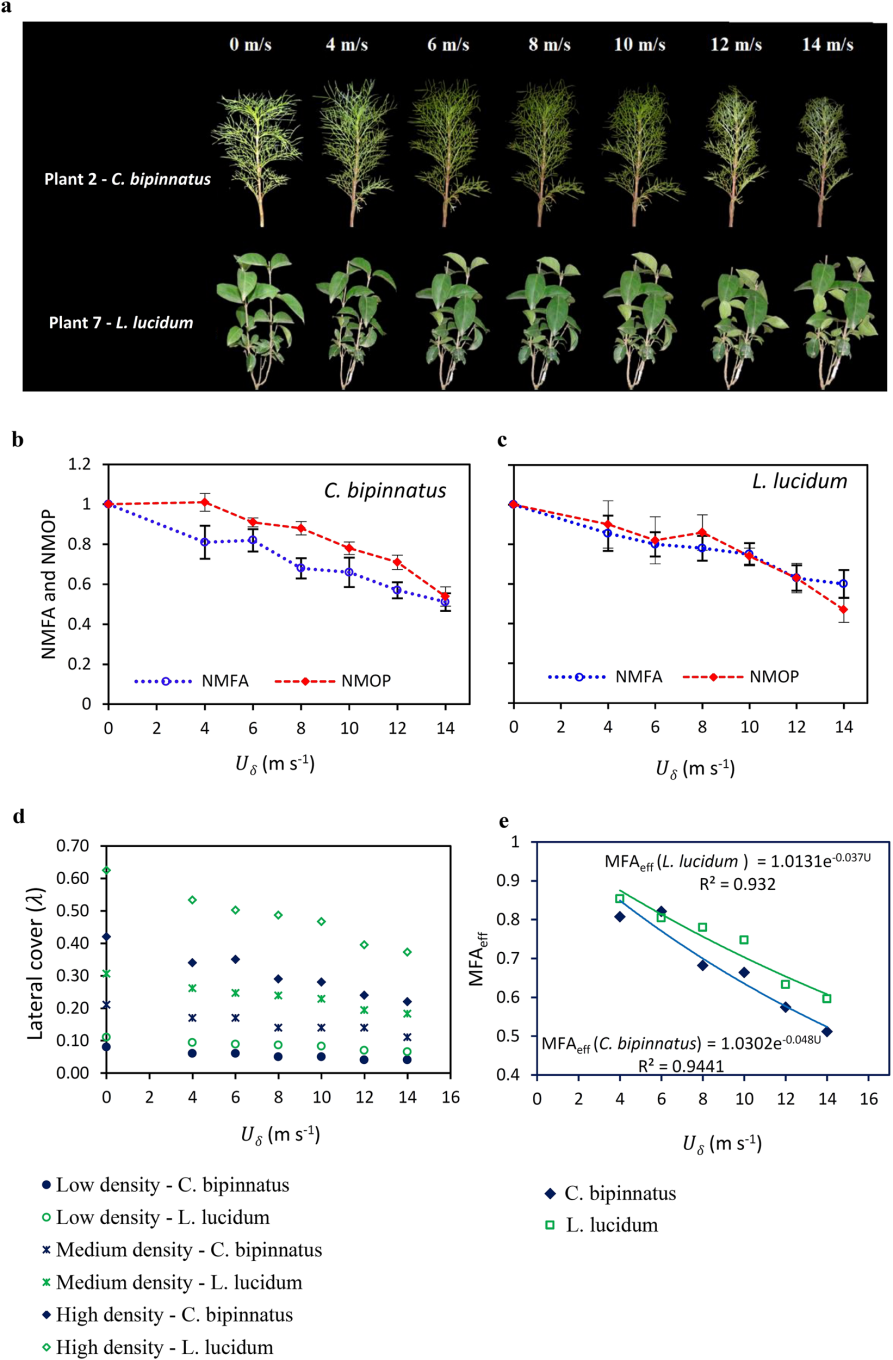
The responses of *C. bipinnatus* and *L. lucidum* to airflow. (**a**) Reconfiguration of plants number 2 of *C. bipinnatus* and number 7 of *L. lucidum* when subjected to wind velocities of *U*_*δ*_ = 0, 4, 6, 8, 10, 12 and 14 m s^−1^. (**b**) Decrease of normalized mean frontal area (NMFA) and optical porosity (NMOP) of *C. bipinnatus* with increasing wind velocity. Vertical bars show the calculated Standard Error of mean. (**c**) Decrease of normalized mean frontal area (NMFA) and optical porosity (NMOP) of *L. lucidum* with increasing wind velocity. (**d**) Decrease of lateral cover (*λ*) with increasing wind velocity in all density cases. (**e**) Exponential decrease of mean frontal area efficiency (MFA_eff_) with increasing wind velocity.

When plants are exposed to wind they respond both morphologically and aerodynamically. The main morphologic responses are reconfiguration and deformation (Fig. 1a and Supplementary Information) which result in a change in aerodynamic characteristics including frontal area (FA), optical porosity (OP), frontal area efficiency (FA_eff_ is FA in still air compared with FA in wind), and frontal area display efficiency (FAD_eff_) (Fig. 1). When wind velocity increases from *U*_*δ*_ = 0 m s^−1^ to *U*_*δ*_ = 14 m s^−1^ both plant types lose their ability to impede airflow. This is reflected in losing a part of their FA (Fig. 1b,c and Supplementary Table S7) and FA_eff_ (Fig. 1e). In addition, lateral cover (*λ*) decreases when wind speed increases (Fig. 1d). However, even though frontal area in both plant types is reduced, their porosity is decreased thereby minimising the amount of airflow passing through them. Plants reduce their porosity by decreasing optical porosity (OP) simultaneously with frontal area (Supplementary Fig. S7d). Furthermore, frontal area display efficiency (FAD_eff_) increases with increasing wind velocity for both plant types (Fig. 1e) which indicates that both plant types tend to reduce permeability by blocking spaces within the canopy. Overall, the exposed area and porosity are critical factors in the response of the plants to airflow.

### Characterizing the shelter effect of the plants

**Figure 2:**
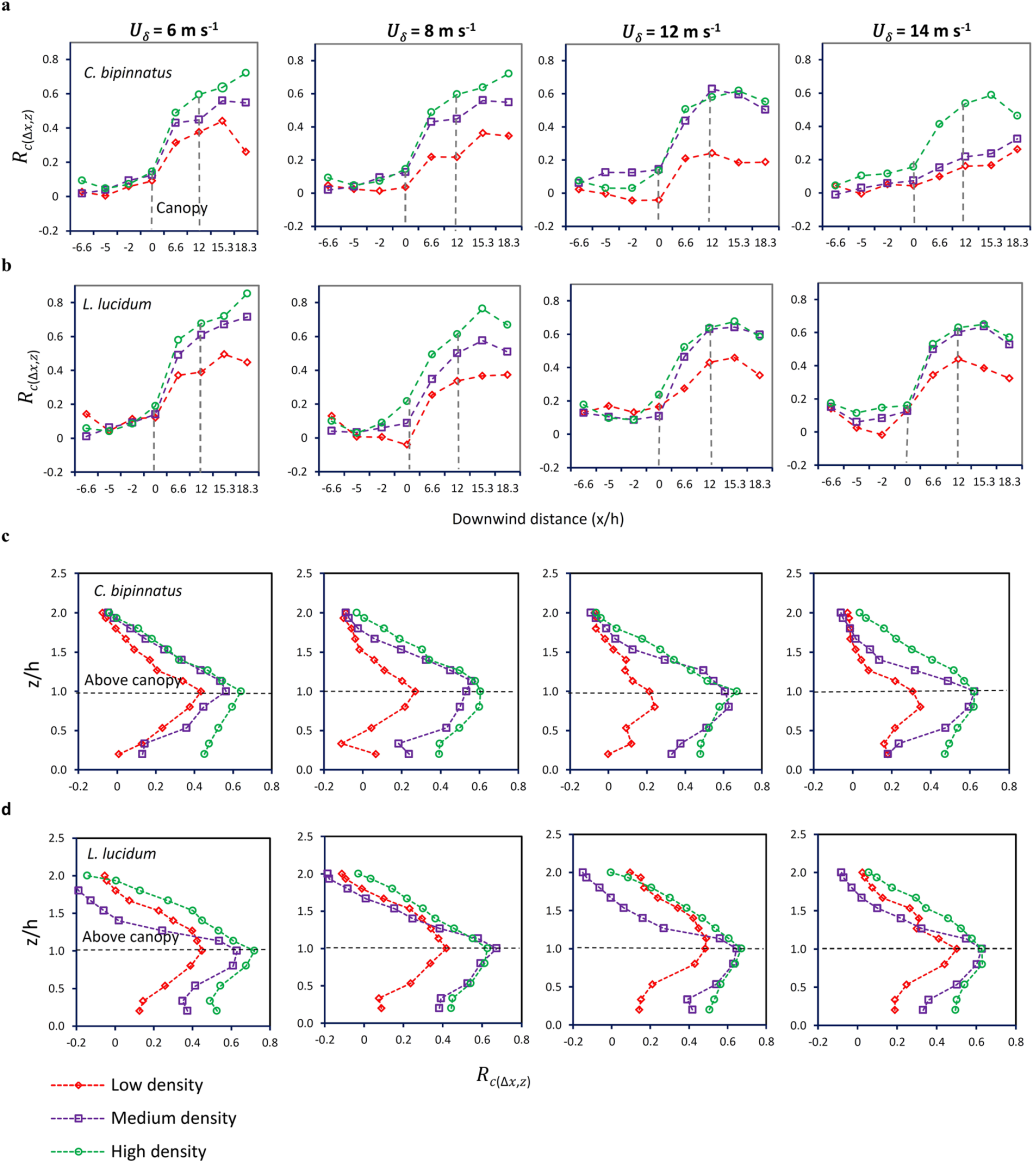
Horizontal and vertical shelter effect of the plants as a function of *λ* (frontal area index) in various wind speeds. (**a**) Horizontal shelter effect of *C. bipinnatus*. (**b**) Horizontal shelter effect of *L. lucidum*. (**c**) Vertical shelter effect of *C. bipinnatus*. (**d**) Vertical shelter effect of *L. lucidum*. The horizontal profiles are plotted at height of *z*/*h* = 0.8 and vertical profiles are plotted at downwind position of *x*/*h* = 12.

We characterize the shelter effect of the plants (*R*_*c*(*x*,*z*)_, wind speed coefficient) as a function of *λ* (lateral cover) (Fig. 2 and Supplementary Fig. S13) and find that the shelter effect of plants is influenced by lateral cover (*λ*). *R*_*c*(*x*,*z*)_ values increase vertically from the surface to the canopy height and horizontally from the leading edge of the canopies to beyond their downwind end for both plant types. Increasing *R*_*c*(*x*,*z*)_ values horizontally are attributed to the reduced wind influence on *λ* as the downwind distance from leading edge of the canopy increases. Increasing foliage concentration with increasing downwind distance results in more spaces being blocked and less airflow passing through the canopy. Highest *R*_*c*(*x*,*z*)_ values are observed horizontally beyond the end of the canopies and vertically within the upper canopy where foliage concentration is greatest.

A difference in vertical and horizontal shelter effect is observed in different densities of both plant types (Fig. 2 and Supplementary Fig. S13). The difference in *R*_*c*(*x*,*z*)_ values between low densities, and medium and high densities, is much more significant than when comparing medium with high density in both plant types. This differential for low density is enhanced under increasing wind speeds, indicating that in higher wind speeds the ability of low-density plant covers to protect the surface and decrease wind velocity decreases substantially. These results provide evidence that in low wind velocities both plant types are able to protect the surface similarly whether planted in low, medium or high densities. In moderate and higher wind velocities, however, plants show a similar efficiency in medium and high densities but their ability is much less at low densities. We attribute these results to plant responses to airflow and suggest that plant streamlining and reconfiguration are critical structural factors determining plant efficiency in different densities. In higher densities individuals are too close to each other due to their greater concentration, and thus plant streamlining and change in aerodynamic characteristics –decreasing FA and FA_eff_ and increasing OP and FAD_eff_ –do not affect the resistance of the plant as much as in lower densities. In low density, the decrease in flow resistance of plants is greater due to high streamlining which reduces more frontal area and lateral cover in the upper canopy compared to medium and high densities. Low density plants thus exert minimum drag on airflow. As a result, maximum airflow is transferred to the lower area within or beneath the canopies. This result is supported by a smaller sheltering effect reported for plants than for rigid wooden cubes, due to plant streamlining at high wind speeds^23^. Furthermore, plant streamlining results in increasingly narrow areas being covered at low densities^23^. The greater influence of *λ* is due to plant leaves fluttering in response to higher wind speed. This behaviour acts as a function opposite to streamlining and increases plant resistance to flow in higher wind velocities^23^. The effect of fluttering is most important at medium and high densities due to the larger numbers of plants present, which results in the canopy exerting greater drag on airflow and absorbing greater momentum.

Our results also provide evidence that the role of plants located at the beginning of the canopy is much more significant than those appearing at the downwind end. Individuals at the leading edge of the canopy are exposed to the greatest load of airflow and are therefore critical in the potential of the entire canopy to influence wind speed. We conclude that using plants with lower reconfiguration and lesser streamlining responses to wind at the beginning of a canopy would promote the potential of the complete canopy to protect the surface both beneath and immediately beyond the planted area.

**Figure 3:**
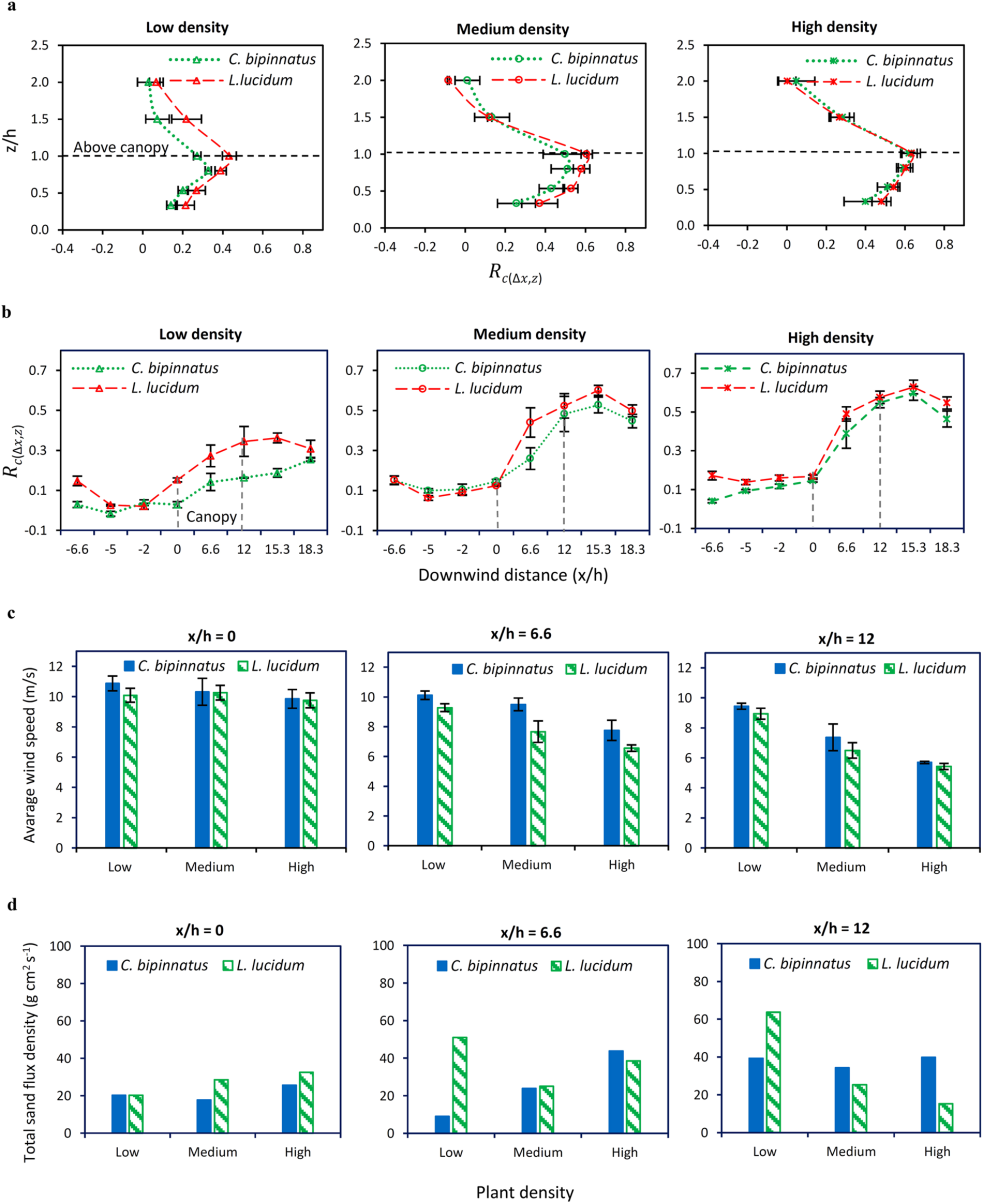
Comparing the efficiency of *C. bipinnatus* with *L. lucidum* in protecting the surface. (**a**,**b**) The vertical shelter effect is obtained by averaging the *R*_*c*(*x*,*z*)_ values at downwind positions of *x*/*h* = 6.6 – 15.3 and the horizontal shelter effect is obtained by averaging the *R*_*c*(*x*,*z*)_ values within a layer of *z*/*h* = 0.3 – 1 (below the canopies’ height) under wind velocity of *U*_*δ*_ = 14 m s^−1^, and compared in different densities. (**c**,**d**) The total sand flux density and the average wind velocity were obtained within a layer of *z*/*h* = 0.2 to *z*/*h* = 0.8 (below the canopies’ height) at each downwind position under wind velocities of *U*_*δ*_ = 14 and 15.5 m s^-1^. Error bars in a,b and c show the calculated Standard Error of mean.

To derive reliable comparisons of the efficiency between the plant types we examine both the vertical and horizontal shelter effect (Fig. 3a,b and, Supplementary Figs S14, S15), the total sand flux and the average wind velocity (Fig. 3a,b).

### Comparing the effectiveness of the two plant types in aeolian erosion control

The results suggest that broad-leaved *L. lucidum* is more efficient than narrow-leaved *C. bipinnatus* in reducing wind velocity due to its greater *R*_*c*(*x*,*z*)_ values (Fig. 3a,b and Supplementary Figs S14, S15) and lower mean wind velocity (Fig. 3c). Sediment flux for *L. lucidum* also decreases more in medium and high densities than for *C. bipinnatus*. The different effectiveness of the two plant types is attributed to their different morphologic and aerodynamic responses. *L. lucidum* is more effective in resisting the wind load through less reconfiguration, and the aerodynamic form of this reconfiguration tends to be less porous, thus exposing a greater projected area to airflow. In general, average frontal area (FA) exposed to the wind and blown particles, and lower porosity (OP), are critical factors which affect a plant’s ability to absorb momentum from airflow and thereby allow less throughflow of wind and windblown particles.

The decrease in key parameters is greater for *C. bipinnatus* than *L. lucidum*: FA (∼40% and ∼30% for *C. bipinnatus* and *L. lucidum *respectively) (Fig. 1b and Supplementary Table S7), *λ *(∼48% and ∼40% for *C. bipinnatus* and *L. lucidum *respectively) (Fig. 1c) and FA_eff_ (decay rate of FA_eff_ ∼0.048 and ∼0.037 for *C. bipinnatus* and *L. lucidum* respectively) (Fig. 1d). OP and FAD_eff_ (Fig. 1a and Supplementary Fig. S7c) decrease more in *L. lucidum*. As a result, a larger projected area of *L. lucidum* is exposed to airflow but the porosity of *C. bipinnatus* is greater. The more optimal aerodynamic response of *L. lucidum* enables it to absorb more momentum allowing the minimum airflow to pass through its canopy, and thus reduce wind speed and decrease the likelihood of sand movement (Supplementary Information). The shape of leaves (*C. bipinnatus* is a narrow-leaved and *L. lucidum* is a broad-leaved plant) and the stiffness of stems, branches and leaves are important morphological components corresponding to the greater resistance of *L. lucidum*.

In contrast to the overall ability of *L. lucidum* to reduce wind speed, at low plant densities the sand flux is greater for *L. lucidum* than for *C. bipinnatus* (Fig. 3d). This result is most likely caused by the observed and morphologically-determined characteristic of plant fluttering, which causes airflow to fluctuate and become turbulent. Such turbulence increases shear stress near the surface which enhances erosion and acceleration of sand particles, especially in low plant density when a higher proportion of the surface is exposed. By maintaining its frontal area and reducing its porosity, *L. lucidum* impedes airflow and reduces wind speed. However, turbulence in the canopy limits the optimal performance of *L. lucidum* in controlling blown sediment in low density. Visual observation shows greater fluttering of *L. lucidum* than *C. bipinnatus* especially at higher wind velocities (*U*_*δ*_ >10 m s^−1^) (Fig. 1a and Supplementary Fig. S6). In medium and high densities, plant drag overcomes the turbulence created and thus the response of *L. lucidum* reduces sediment flux. However, in low density, because the quantity of plants in the canopy is low and wind flows easily between the individual plants, fluttering is more pronounced. Fluttering thus reduces drag and influences the ability of *L. lucidum*, compared to *C. bipinnatus*, to affect windblown sediment.

The influence of turbulence is indicated by a decrease in fluttering with downwind distance within the canopies. As a result of the increased downwind distance and enhanced influence of lateral cover (*λ*) (Fig. 2), drag dominates over turbulence at the downwind end of the canopy. This decreased influence of turbulence explains the smaller difference in sand flux density between the two plant types at low densities, and the greater difference with medium and high densities. We conclude that it takes a certain distance before plants exert the maximum reduction in wind speed through drag, and thus for minimising the potential for sediment transport and surface erosion. Beyond this distance plants show their ability to influence sediment transport. The evidence presented suggests that the difference between plant species in sheltering the ground surface and reducing sediment transport can be observed at the immediate downwind end of a canopy.

The significant difference apparent between the two plant types in low density (Fig. 3) suggests that plants with different morphologies are likely to present similar efficiencies in influencing the sediment transport system in higher densities but substantial differences in lower densities. With this result and those from Fig. 2 we are the first to identify a strong relationship between plant morphologic and aerodynamic characteristics and erosion-reducing efficiency, and suggest that the function of these characteristics is critical at low plant densities and that the flow resistance of individual plants determines the efficiency of the canopy. In drylands low-density cover is a characteristic feature^7^ and intensive wind is predominant in producing dust emission^1,32^. In such areas, selecting plants for re-vegetation projects in order to facilitate aeolian erosion control should be focused on species which reconfigure least in response to wind and present low porosity, high frontal area and less deformation.

Consistent with previous studies, we strongly support the critical role of vegetation in controlling aeolian erosion^4,6–13^. However, we incorporate plant morphologic and aerodynamic characteristics with plant density and nominate them as a critical combination of parameters that affect aeolian erosion. Attributing a plant’s airflow-response to its morphology and linking it to the plant’s efficiency in protecting the surface is a critical concept, which is particularly important for selecting the most efficient plants for effective erosion control. Wind-resistant vegetation that presents low porosity, high frontal area and less deformation is highly recommended for providing maximum ground surface protection and thus producing the most efficient barriers to aeolian erosion. The resistance of vegetation is particularly important in low-density covers. Plant morphological characteristics combined with plant density need to be evaluated for effective control of aeolian erosion, following which other parameters such as plant height and distribution patterns need to be assessed.

## Methods

All experiments were carried out in the wind tunnel of the Key Laboratory of Environmental Dynamics on the Loess Plateau, at the Shanxxi Normal University in Xi’an, China (Supplementary Information). The experimental section was 700 cm long, 50 cm wide and 60 cm high and was adequate for our experiments.

### Selecting the plants

The aim of this study was to compare two plant types with different vegetative morphology, so selecting these plants was an important first stage of the research. *C. bipinnatus* and *L. lucidum* (Supplementary Fig. S5) were chosen in accordance with the aim of the study (Supplementary Information). Plants with a height of about 15 cm were transferred to trays where they were distributed in regular staggered rows in high, medium and low-density configurations following the same overall planting design patterns (Supplementary Fig. S3). The position of each plant in the array was determined by carefully marking the location for each roughness array configuration to provide the proper inter-plant spacing to achieve the target *λ* values.

### Obtaining the density of the plants

Plant density was quantified using two parameters, namely, the horizontal vegetation cover (*C*_*v*_)^33^ and the frontal area index or lateral cover (*λ*)^34^. *C*_*v*_ was calculated as *N*.*A*_*PV*_/*A*_*T*_ and *λ* was calculated as *N*.*A*_*FV*_/*A*_*T*_, with *N* being the number of plants in the canopy (*N* had three different values corresponding to the three different densities of vegetation cover), *A*_*PV*_ is the averaged plan view area of vegetation elements, *A*_*FV*_ is the averaged frontal view area of the plants and *A*_*T*_ is canopy area in the wind tunnel (10,000 cm^2^) for both plants at all densities. Digital image processing and ArcGIS were used to obtain the averaged frontal area (*A*_*FV*_) and plan view area (*A*_*PV*_) values. Seventy-five plants of *C. bipinnatus* and 102 plants of *L. lucidum* were available for sampling. Plant numbers 2, 9, 16, 23, 30, 37, 44, 51, 58, 65 and 72 for *C. bipinnatus* and numbers 5, 15, 25, 35, 45, 55, 65, 75, 85, 95 and 105 for *L. lucidum*were drawn from systematic sampling of each population. A new ID number (from 1 to 11) was defined for each of the eleven individual plants of each type. This sample was used to derive the average plan view area (Supplementary Figs S8, S9) and frontal area (Supplementary Figs S10, S11) of plants at their different densities. Both *C. bipinnatus* and *L. lucidum *have horizontal cover of *C*_*v*_ ∼ 10%, 28% and 56% in low-, medium- and high-density configurations respectively. *C. bipinnatus *have frontal area indices of *λ* ∼ 0.08, 0.22 and 0.44 and *L. lucidum λ* ∼ 0.11, 0.30 and 0.61 in low-, medium-, and high-density configurations respectively.

### Calculating optical porosity

Pore identification (canopy openness) in images is a difficult part of estimating pore area, especially near the outer edge of plants because a pore has a three-dimensional area but appears with a two-dimensional structure^35^. Several rules have been used to define pores of plants^35^, involving different methods for different plants, with only two pore types being defined (pore type 1 and pore type 2). An additional pore type, pore type 3, was devised in this study, as existing procedures did not include spaces between leaves and stems as a pore. Rules identifying the three pore types are as follows: Pore Type 1: the parts of the plant that formed a closed object in which light can pass through the plant; Pore Type 2: where a zone between the two closest leaves near the plant edge (or leaflet or branches) was less than a quarter of the total width of the partially leaf enclosed area; and Pore Type 3: where an area between the two closest leaves (or leaflet or branches) near the plant edge was more than a quarter of the total width of the partially leaf-enclosed area and can be assumed as a space allowing throughflow (Supplementary Fig. S12). After creating a polygon around the plants, the images were reclassified into two categories: plant material and pore. The area covered by individual raster cells was calculated using the scale included in each photograph. Optical porosity was calculated by the following equation^35^:

1 $$OP=\frac{(total\,pore\,area)}{(total\,solid\,plant\,area)+(total\,pore\,area)}$$

To maximize the precision of defining pores the same person analyzed all images.

### Subjecting the plants to wind and obtaining their frontal area and optical porosity

*C. bipinnatus* and *L. lucidum* were exposed to different wind velocities and their frontal area (frontal area efficiency and efficiency of frontal area display), pore area and optical porosity were estimated using an image processing procedure. The samples of eleven plants of each type were divided into two categories based on frontal area. Within each category four plants were selected based on their frontal areas lying in the highest, middle and lowest range of measured plant frontal areas. The four selected plants (numbers 3, 6, 7 and 10 of *C. bipinnatus* and numbers 5, 6, 7 and 10 of *L. lucidum*) were used as representative of all plants of their type and placed in the wind tunnel to be subjected to different wind velocities. To obtain images of plants, a high-resolution digital camera with strong flashlight was placed at a distance upwind of the plants in the middle of the wind tunnel floor (y = 25 cm). The flashlight was strong enough that internal illumination of the wind tunnel was not necessary. Different locations were tested to find an appropriate place to minimize the influence of the camera on airflow (it might act as an obstacle) and maximize the resolution of photos. Wind velocity was measured by a Pitot tube installed beside the plants. A graduated metal rod was placed in the soil beside the plants to provide a reference size of the object in the acquired images (Supplementary Fig. S12). For this study frontal area efficiency (FA_eff_) (the amount of frontal area projected towards the wind direction as a fraction of total frontal area; total frontal area represents the plant in still air) and frontal area display efficiency (FAD_eff_) (the ratio of mean frontal area to mean pore area) were devised and calculated as follows:

2 $${{\rm{FA}}}_{{\rm{eff}}}=\frac{{{\rm{FA}}}_{{\rm{w}}}}{{{\rm{FA}}}_{{\rm{sa}}}}\,$$

where FA_w_ is frontal area projected towards the wind direction and FA_sa_ frontal area of the plant in still air (total frontal area).

3 $${{\rm{FAD}}}_{{\rm{eff}}}=\frac{{\rm{MFA}}}{{\rm{MPA}}}\,$$

where MFA and MPA are mean frontal area and mean pore area respectively.

### Measuring wind velocity and sand flux density

At eight points pitot-tubes were installed to monitor the wind profiles at twenty heights: *z* = 3, 5, 8, 12, 15, 17, 19, 21, 23, 25, 27, 29, 30, 32, 34, 36, 38, 40, 42 and 44 cm, on a line displaced by y = 25 cm from the centreline of the test floor (Supplementary Fig. S1c and Supplementary Table S2). At four points sandsamplers (WITSEG samplers) were set up to measure sand flux density (each sampler is sectioned into fifteen sand chambers of having 2 × 1 cm openings to collect the blown sediments at height increments of 2 cm from 0–2 cm to 28–30 cm) in all configurations of *C. bipinnatus* and *L. lucidum* and over bare sand (Supplementary Fig. S1c Supplementary Table S3). Freestream wind velocity of 15.5 m s^−1^ was applied to measure blown sand flux in all configurations. The duration of constant wind velocity was 300s. This duration was applied to yield a sufficient amount of sand in the sampler without overloading it and to reduce plant damage from sand bombardment. The mean diameter (−log_2_ *d*) of the sand used for the experiments was 0.18 mm (2.42 *ϕ*), and the sand is well sorted (standard deviation 0.41, skewness 0.05, and kurtosis 1.02). The threshold velocity of the sand at the centreline of the wind tunnel was observed to be 5 m s^−1^. About 84% of the sand lies within the 100–250 μm range, which is predominantly within the sand range for saltation^36^ and corresponds to general values for blown sand^37^. The sands are therefore highly vulnerable to wind erosion^38^ (Supplementary Information and Supplementary Table S4).

We evaluated the efficiency of the plants in sheltering the surface and reducing wind velocity by using a dimensionless wind reduction coefficient (*R*_*c*Δ*x*,*z*_, Cornelis and Gabreils, 2005), as follows:

4 $${R}_{c{\rm{\Delta }}x,z}=1-(\frac{{u}_{{\rm{\Delta }}x,z}}{{u}_{0{\rm{\Delta }}x,z}})$$

where *R*_*c*Δ*x*,*z*_ is the wind reduction coefficient, Δ*x* is the distance from the windbreaks (in windbreaks height *H*), *z* is the height above the surface (in windbreaks height *H*), *u*_Δ*x*,*z*_ is the time-averaged wind velocity disturbed by the windbreaks (m s^−1^), and *u*_0Δ*x*,*z*_ is the time-averaged wind velocity in the absence of a windbreaks (m s^−1^). In general, as *R*_*c*Δ*x*,*z*_ increases the shelter effect of the plants increases. The elevations (*z*) and downwind distances (*x*) were also normalized by mean canopy height (*h* = 15 cm) in all configurations as *z*/*h* and *x*/*h* (where *z* is the height of measured wind velocity, *h* is the canopy height and *x* is the distance from the leading edge of canopy).

## Electronic supplementary material

Supplementary Information
