# Supplementary material for: Vegetation morphologic and aerodynamic characteristics reduce aeolian erosion
Source: Sci Rep. 2017 Oct 9;7:12831. doi: 10.1038/s41598-017-13084-x (PMC5634502; doi:10.1038/s41598-017-13084-x)
Supplement: Supplementary file 1 — Supplementary Information [file 41598_2017_13084_MOESM1_ESM.pdf]

Vegetation morphologic and aerodynamic characteristics reduce aeolian erosion

Abbas Miri[1,2*], Deirdre Dragovich[2], Zhibao Dong[3]

[1]Department of Watershed and Range Management, Faculty of Water and Soil, University of Zabol, Zabol, Iran.[2]School of Geosciences F09, University of Sydney, Sydney, NSW 2006, Australia. [3]Key Laboratory of Desert and Desertification, Cold and Arid Regions Environmental and Engineering Research Institute, Chinese Academy of Sciences, Lanzhou 730000, Gansu, China. *email: amir5402@uni.sydney.edu.au

1. Conducting the wind tunnel experiments

All experiments (81 experiments were done in this study, Supplementary Table S1) were carried out in the wind tunnel of the Key Laboratory of Environmental Dynamics on the Loess Plateau, at the Shanxxi Normal University in Xi'an, China (Supplementary Fig. S1a shows wind tunnel schematically). The wind tunnel is a 'blowing-type' which is specifically designed for aeolian erosion studies, and therefore is equipped to contend with blowing sand particles. The working section of the tunnel has dimensions of 50 cm wide, 60 cm high and 700 cm long, in which the flow speed can be controlled from 0.1 to 30 m s^{-1}.

Prior to experiments, wind profiles were measured as a function of distance along the tunnel without plants to select the appropriate locations to set up vegetation and Pitot tubes. Eight points were included to monitor the wind regime to better understand the protective possibilities of vegetal barriers. The points of measurement are shown in Supplementary Fig.S1c and listed in Supplementary Table S2. The interval distance between Pitot tubes 5 and 6 and plants upwind in the canopy was considered carefully. Since the goal was to understand the wind regime in a canopy rather than determining the effect of individual plants on airflow, the Pitot tubes were installed in an appropriate position to avoid airflow perturbations generated by the short-lived motions of the individual stalks and leaves of the upwind sheltering plant. In cases where a plant upwind of the Pitot tubes was too close to the Pitot tube and it seemed that flow could be affected directly by the plant, the plant was removed. To maximize the accuracy of data collected the end of the tube was carefully oriented to directly face the incoming airflow.

To decrease the error in sand measurements the spacer between the openings is made very thin. The leading part of the sampler is wedge-shaped therefore the width of openings is less than the chambers so that the probability of the sand sampler interfering with airflow is minimised. To reduce the air pressure and increase the sand-catching efficiency to more than 90%, a screened vertical vent is connected to each sand chamber. Sand samplers 2 and 3 were placed in a position to

ensure that the individual stalks and leaves of the upwind sheltering plants had no effect. Similar to the setting up strategy of Pitot tubes where the plants upwind of the sampler were too close to the sampler and it seemed that flow could be affected, the plants were removed, causing a minimal or at least undetectable effect on wind speed between consecutive sampler positions. The samplers were set into the sand in the middle of the wind tunnel floor, so that the bottom of the lowest opening of the sampler was flush with the sand surface. Four locations were included to measure vertical and horizontal sand flux along the wind tunnel. The location of sand samplers is shown in Supplementary Fig.S1c and summarized in Supplementary Table S3.

Supplementary Fig.S2 illustrates Pitot tube and sand samples set-up in experiments with *C. bipinnatus* and *L. lucidum* in high, medium and low densities.

The sand used for the experiments was collected from sand dunes located in the Shapotou area, southeast of the Tengger Desert in China. The sands were wet because they were collected in spring. They were dried completely in an oven model DHG-9203A. In some samples, litter was observed and to remove this, the sands were sieved. In all experiments the floor of the wind tunnel was covered with sand to a depth of 3 cm, starting from downwind of the artificial roughness field. In sand flux monitoring experiments, sand in the bare surface area (upwind of the canopy) was eroded and had accumulated on the surface within the planted area and at the downwind portion of the work section. After each test the sand in the unplanted surface section was renewed to provide consistent surface conditions, and the sand accumulated in the canopy and on the floor beyond the canopy was removed completely and replaced with dried sand.

For setting up the vegetation, a series of three aluminium trays was placed in appropriate positions within the tunnel. The depth of the trays was 10 cm in order to allow plants to develop their roots. The plants with a height of about 15 cm (with standard deviation about 1 cm) were formed in three different levels of canopy density for *C. bipinnatus* and *L. lucidum* of low-, medium and high-density configurations (Supplementary Fig. S3) followed the same overall planting design patterns.

Brown *et al.*[1] and Webb *et al.*[2] indicated that the configuration of roughness elements is an important factor affecting roughness sheltering effect, the drag partition, and sediment transport. However, in accordance to the aim of the study (comparing the morphologic and aerodynamic responses of the two types of plant to airflow in the same condition and linking these to their sheltering potential) the plants were distributed in regular staggered rows in all experiments. This staggered pattern has been used in many wind tunnel studies for artificial roughness[3-7] and live vegetation roughness[8-11]. The position of each plant in the array was determined by carefully marking the location for each roughness array configuration to provide the proper inter-plant spacing to achieve the target λ values.

The plants were transferred to the trays and the trays were left outside the wind tunnel for 48 hours to give enough time for the plants to develop their roots in the soil and become stable enough for experimentation. The trays were then transferred to the wind tunnel. The trays were aligned in designated positions in the wind tunnel. The trays created a vegetation cover (canopy) with dimensions of 200 cm length × 50 cm width and an area of 10,000 cm^2 in total. Supplementary Fig.S1b represents the location of vegetation canopies in the work section of the wind tunnel.

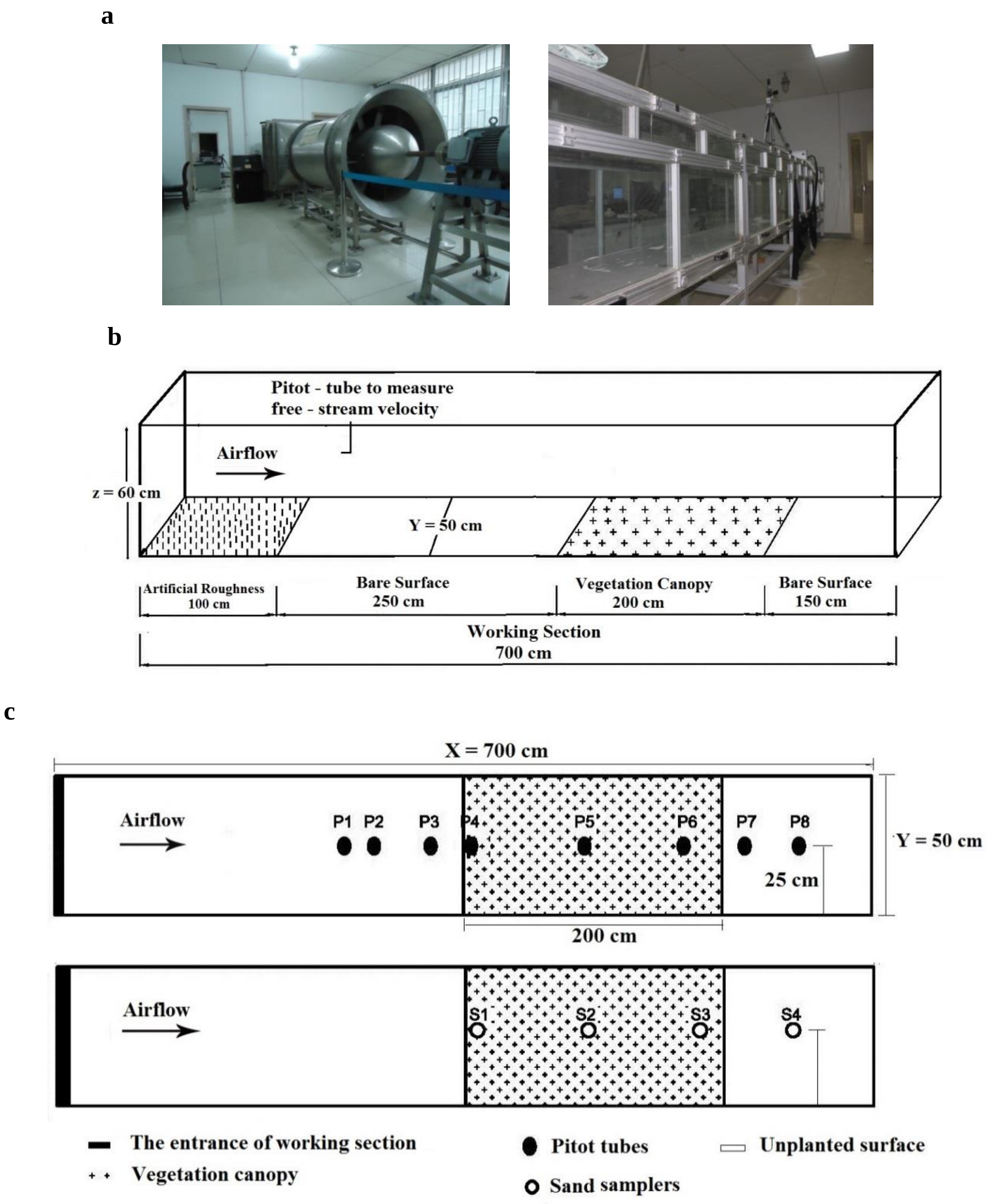

Supplementary Fig. S1: Overview of the wind tunnel and set up of instruments and vegetation. (a) Schematic diagram of the wind tunnel. (b) Layout of the vegetation canopy position in the work section of the wind tunnel. (c) Schematic locations of Pitot-tubes and sand samplers in work section of the wind tunnel. The location of pitot tubes and samplers are as listed in Suppl. Tables 2 and 3.

Supplementary Table S1
Summary of the experiments (Exp) and sub-experiments (in total 81 experiments were done in this study)

	Number of experiments and sub-experiments		
	**Exp1:** Plants subjected to the wind	**Exp2:** Wind velocity profile monitoring	**Exp3:** Sand mass flux profile monitoring
C. bipinnatus	24	12	3
L. lucidum	24	12	3
**No Plant**	0	2	1
**Total**	**48**	**26**	**7**

Supplementary Table S2

The locations of Pitot tubes in the work section of the wind tunnel

Pitot tube	Location	Downwind distance (cm)
**P1**	100 cm upwind of the canopy (150 cm downwind from the leading edge of the roughness field)	$x = -100$
**P2**	75 cm upwind of the canopy (175 cm downwind from the leading edge of the roughness field)	$x = -75$
**P3**	30 cm upwind of the canopy (220 cm downwind from the leading edge of the roughness field)	$x = -30$
**P4**	On the leading edge of the vegetation (250 cm downwind from the leading edge of the roughness field)	$x = 0$
**P5**	100 cm downwind from the leading edge of vegetation (In the middle of the vegetation canopy)	$x = 100$
**P6**	180 cm downwind from the leading edge of vegetation	$x = 180$
**P7**	230 cm downwind from the leading edge of vegetation (30 cm downwind of the canopy)	$x = 230$
**P8**	275 cm downwind from the leading edge of vegetation (75 cm downwind of the canopy)	$x = 275$

Supplementary Table S3

The location of sand samplers installed in the wind tunnel

Sampler	Location	Downwind distance (cm)
**S1**	On the leading edge of the vegetation (350 cm downwind from the entrance of the working section of the wind tunnel)	$x = \text{o}$
**S2**	In the middle of the canopy (450 cm downwind from the entrance of the working section of the wind tunnel)	$x = 100$
**S3**	180 cm downwind from the leading edge of vegetation (530 cm downwind from the entrance of the working section of the wind tunnel)	$x = 180$
**S4**	100 cm downwind the canopy (650 cm downwind from the entrance of the working section of the wind tunnel)	$x = 300$

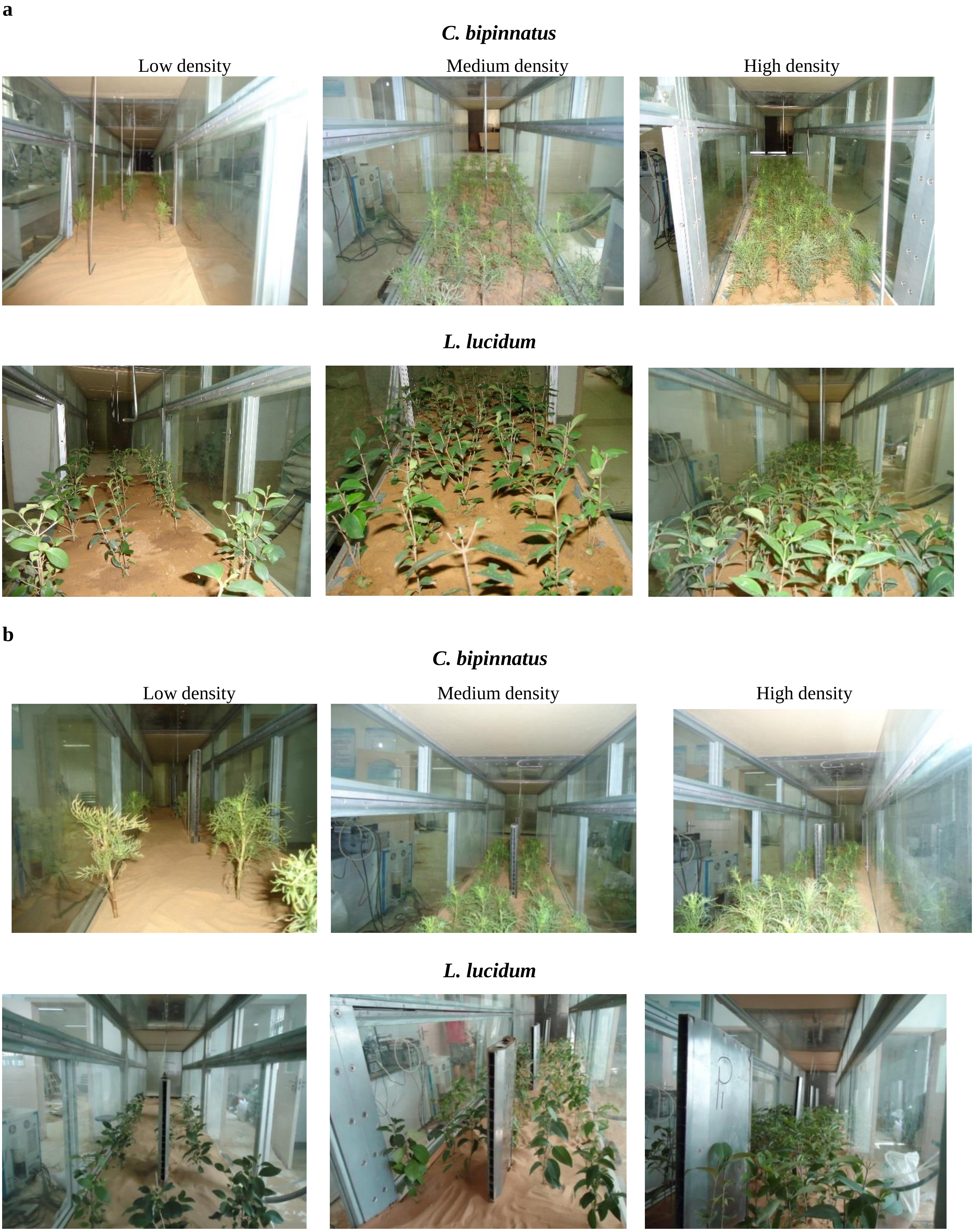

Supplementary Fig. S2: Setup of instruments in the canopies. (a) Pitot tubes setup in low, medium and high density canopies of *C. bipinnatus* and *L. lucidum*. (b) Sand sampler setup in low, medium and high density canopies of *C. bipinnatus* and *L. lucidum*.

Supplementary Table S4

The texture of sands used in the wind tunnel experiments

**Fraction (μm)**	< 50	50 – 100	100 – 250	200 – 500	500 – 1000
**Percentage**	0	2	84	14	0

a

Low density Medium density High density

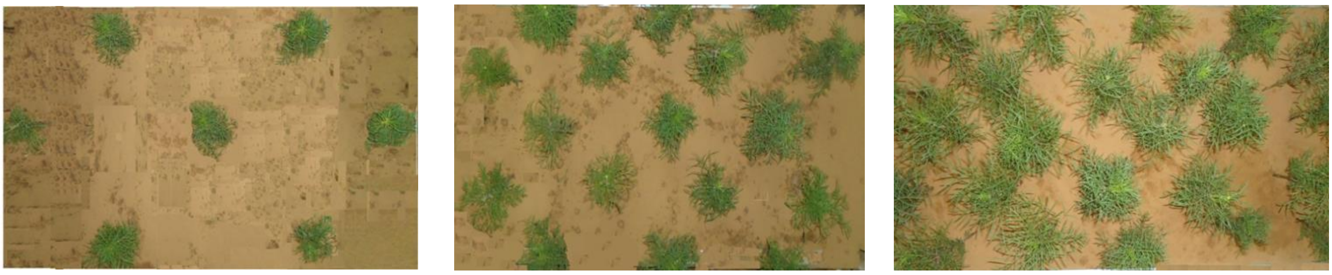

b

Low density Medium density High density

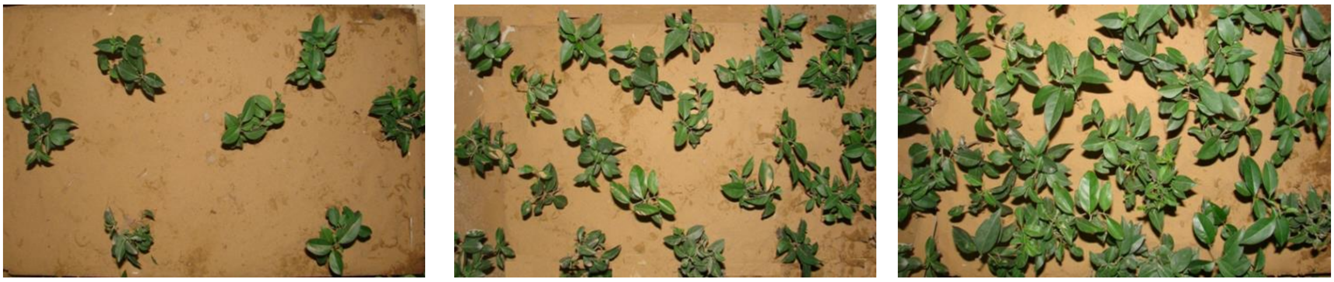

Supplementary Fig. S3: Plant patterns in the wind tunnel experiments in low-, medium- and high-density configurations. (a) *C. bipinnatus*. (b) *L. lucidum Ait* in the wind tunnel.

2. Selecting the plants

A wide study was done about characteristics, germination, growth period, habitats and water requirements of different plant types, especially those growing in dry lands, to select suitable plants to use in the research. Sistan region in the southeast of Iran was visited as a prominent area in which plants are resistant to dry conditions (xerophytic plants)[12]. Initially, nine different plants – *Atriplex leucoclada, Atriplex lentiformis, Atriplex halimus, Atriplex canescens, Peganum harmala, Bromus*

tomentollus, Salsola sp, Salsola regida and *Cardaria draba* – were considered in order to select two plants that were the most suitable to use in the project. In addition to differences in structure, seeds of the chosen plants had to be available for use in experimentation. The seeds were then sourced from a seed production centre in Iran, the Pakan Bazr Isfahan Company, which prepares healthy certified seeds with high viability.

Seeds were transferred to China, planted in labelled baskets, and placed in a greenhouse under controlled conditions and a in small covered garden outside (because of strong sunlight during the day and low temperatures at night at that time of year, the garden was covered by a permeable protective shelter) (Supplementary Fig.S4) at Shaanxi Normal University, China. The seeds were watered and covered with plastic sheeting to reduce evaporation losses and retain soil moisture, thereby providing optimum germination conditions. To test the resistance of the plants to wind and sand bombardment a number of seedlings were selected when they reached about 10 cm tall and transferred to the wind tunnel. Because the plants were gramineous they were too weak to withstand wind and suffered severe damage under wind and sand bombardment. Therefore, they seemed not suitable to use in the wind tunnel tests and all of them were ignored. New criteria were applied to select two plants (*C. bipinnatus* and *L. lucidum*) as follows: (a) *Shape and morphology of the plants:* As plants with different morphology and geometric characteristics have different effects on wind speed, they also have different effects on sediment transport[13,14]. The basic requirement for plant selection was that of two plants with different morphologies and structures. Different leaf shape and branch structure were the required criteria in selecting plants. (b) *Flexibility*: Flexibility is another important characteristic of plants that enhances the complexity of the vegetation resistance and the turbulence of airflow[15], and therefore influences wind speed and sediment transport[16,17]. When a plant is exposed to the wind, its frontal area changes in accordance with wind fluctuations and as a result causes changing roughness density (λ). Because it is difficult to measure how roughness density changes in response to wind conditions, using plants that are not very flexible was desirable. (c) *Availability and growth rate:* Seeds or plants suitable for use in experiments need

to be available. Growing some plants is difficult and it takes a long time before they reach an appropriate size to be used in the wind tunnel. For this research, plants had to be available for transfer to the wind tunnel within a short period, so fast-growing plants needed to be selected for the experiments. (d) *Plant durability and resistance to wind and sand bombardment:* The plant should be strong enough to withstand airflow and sandblasting when exposed to strong wind (more than 8 m/s) and sand flux tests. Strong winds can cause plants to sway excessively, pulling and tugging on their roots, and physically damage plants by breaking them. (e) *Resistance to short-term dry conditions:* To prevent the influence of humidity of the atmospheric air from soil-derived water vapour, watering ceased from the commencement of the experiment and this dry period lasted for several days. Plants used in the tests therefore needed to be resistant to dry conditions during experimentation.

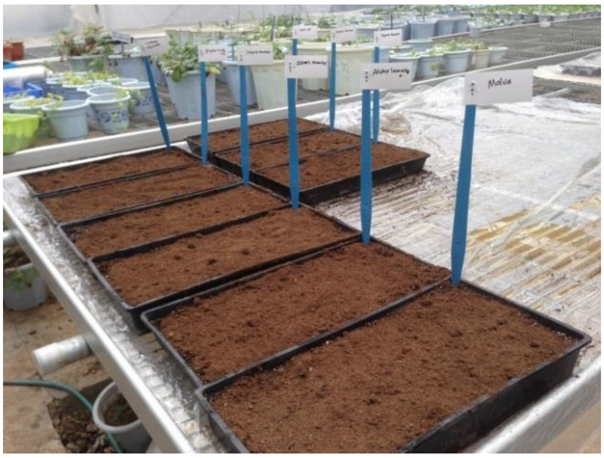

a

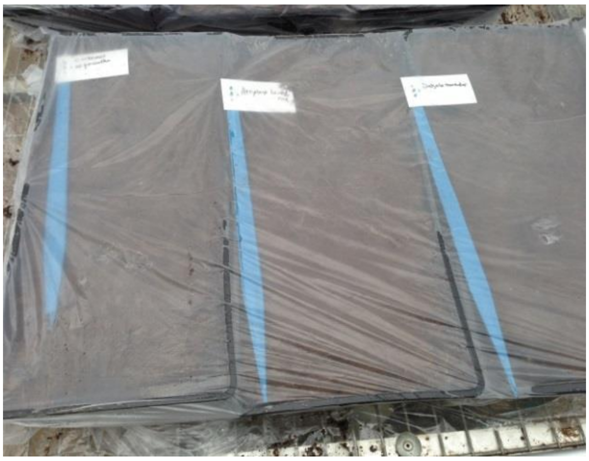

b

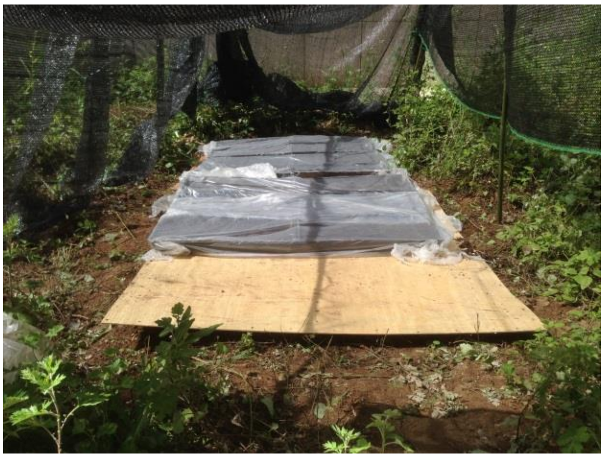

c

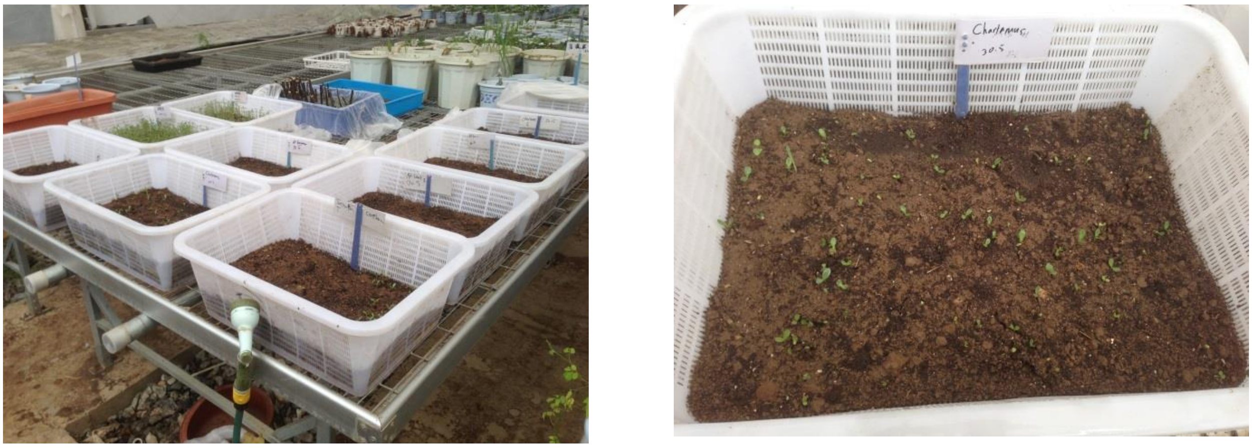

d

Supplementary Fig.S4. Cultivating the plants inside and outside of the greenhouse: (a) Seed boxes; (b) seed boxes covered by plastic sheeting in the greenhouse and (c) in the garden outside in Shaanxi Normal University in China
(d) Seed germination

3. Morphologic characteristics of the selected plants

C. bipinnatus is a medium-sized flowering herbaceous medicinal plant[18] belonging to the *Asteraceae* or *Compositae* family which came originally from Arizona and central Mexico[19].

L. lucidum Ait (common name: broad leaf privet) is a broadleaf tree (Dicots) native to eastern Asia (i.e. China, Korea and Japan) and naturalised in southern Africa, temperate Asia, New Zealand, Hawaii, southern and eastern USA, and southern South America. It is commonly planted in warm regions often for ornamental purposes[20]. In the present study we used saplings of *L. lucidum* in our experiment.

In Supplementary Fig. S5a,b *C. bipinnatus* and *L. lucidum Ait* are shown. The plants present different morphologies and they have different leaves, and branch and peduncles structure. The significant difference between the two plants is in their leaf shape with *C. bipinnatus* exhibiting narrow leaves (Supplementary Fig. S5a) and *L. lucidum* broad leaves (Supplementary Fig. S5b). *C. bipinnatus* is a multi-stemmed plant with leaves that are narrow, pointed and compound (multi-pinnate), deeply cut into threadlike segments and made up of two or more discrete leaflets opposite (there are two leaves per node along the stem). *L. lucidum* has pointed oval-shaped leaves (lanceolate) that are arranged on opposite sides of the stems (opposite pairs). The leaves typically fold upward, like a V, from the mid-vein. The stem of *C. bipinnatus* is erect to ascending, terete, glabrous or sparsely short-pubescent. In contrast to the branch architecture of *C. bipinnatus* that displays only a single vegetative axis, the embryos of *L. lucidum* display a more complex architecture consisting of several axes, one derived from one point by a repetitive process known as branching. The trunk is erect or more usually multiple, and is surmounted by a rounded or vase-shaped crown, often with ascending or spreading branches[21].

a

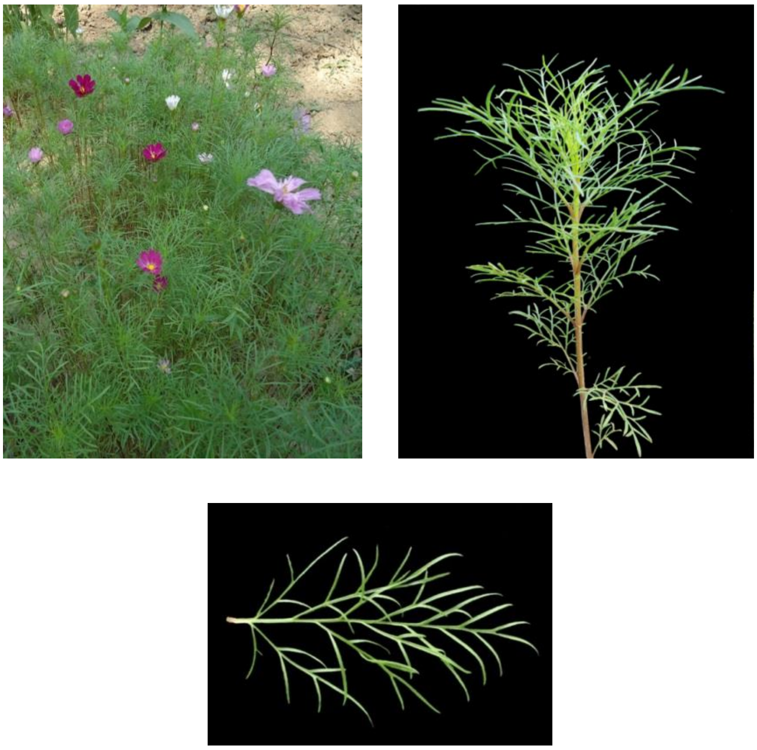

b

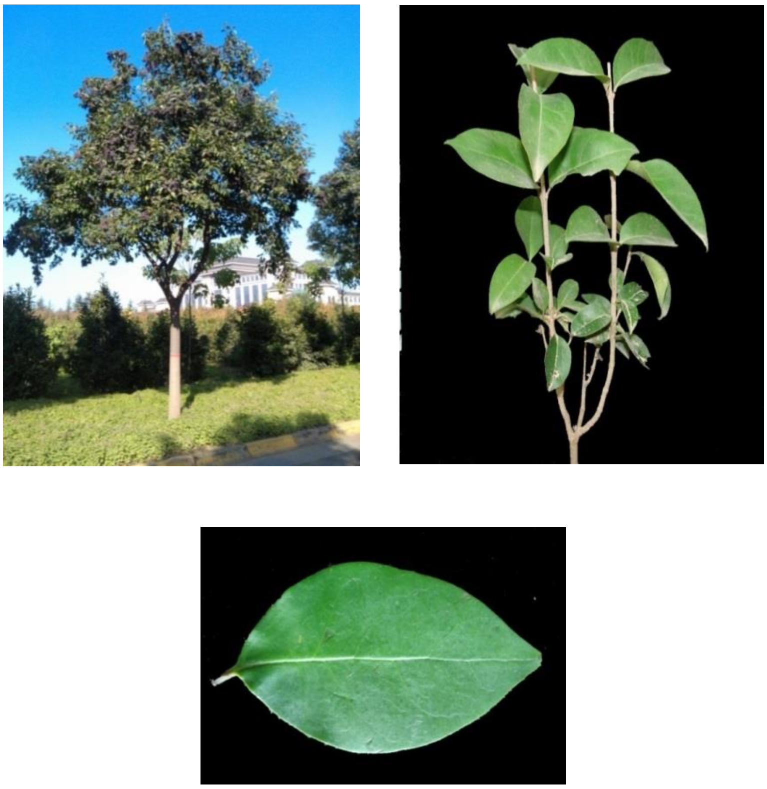

Supplementary Fig. S5 | Morphology of *C. bipinnatus* and *L. lucidum Ait*. (a) *C. bipinnatus* and its leaf shape. (b) *L. lucidum* (tree and seedling) and its leaf shape.

4. Morphologic and aerodynamic responses of *C. bipinnatus* and *L. lucidum* when exposed to a range of wind velocities

Reconfiguration and streamlining are the main morphological response of both plant types to wind which reduces the force of wind and more importantly makes use of flexibility to absorb momentum from airflow by modifying their shape (Supplementary Fig. S6). At lower wind velocities, plant leaves and stems are aligned perpendicular to the flow thereby absorbing the maximum momentum, which reaches a peak at a critical wind speed when plant exposure to wind is decreased. When flow loading is increased plants bend and twist, and the uppermost parts of stems and leaves change orientation leeward, resulting in the appearance of two reconfiguration mechanisms: reduced projected area perpendicular to the flow, and increased streamlining.

The crucial factor that determines the resistance of plants is their drag, which is determined by the surface area exposed to airflow. Orientation of leaves and their role in exerting drag on airflow is important. Comparison of the morphological responses of *C. bipinnatus* and *L. lucidum* reveals that *L. lucidum* maintains its surface area (leaf orientation) in a response which captures the maximum airflow compared with *C. bipinnatus* (narrow leaved plant). *L. lucidum* reconfigures in a manner where its dorsal surface is mostly exposed to the flow in low to moderate wind velocity and at high velocities its ventral surfaces begin flapping and thrashing. *C. bipinnatus* streamlines in the flow direction when wind speed increases, thereby becoming more compact at higher wind velocity, which minimizes its exposed surface area.

Although both plants reconfigure and streamline in higher wind velocities (Fig 1. and Supplementary Fig. S6.) *L. lucidum* responds more effectively in blocking airflow and reducing wind speed. To further support our conclusions we plotted the normalized values of mean frontal area (NMFA) and normalized mean optical porosity (NMOP) against wind velocity for both plant types in Supplementary Fig. S7a and b. By comparing the curve of NMFA for *C. bipinnatus* with that for *L. lucidum,* it appears that the curve of NMFA for *L. lucidum* is higher than that of *C. bepinnatus*. Conversely, comparing the curve of NMOP for *C. bipinnatus* with that for

L. lucidum shows that the curve of NMOP for *L. lucidum* lies lower than that for *C. bipinnatus* over the range of wind speeds tested. Greater values of NMFA and lower values of NMOP for *L. lucidum* in all wind velocities reflect the different behaviour of *L. lucidum* in aligning its body to the wind and decreasing its porosity. *L. lucidum* maintains a higher frontal area and lower porosity than *C. bipinnatus* in all wind speeds.

In the response of plants to wind the decreasing values of OP compared to FA in corresponding wind speeds is also another important function of plants which influences their ability to limit the wind to pass through the canopy. With increasing wind speed, the mean values of FA and OP were observed to be close in *L. lucidum* and far apart in *C. bipinnatus* (Fig. 1b). Although *C. bipinnatus* maintained its frontal area at a higher level in some wind speeds, it was unable to reduce its porosity to lower levels. This inability of *C. bipinnatus* to decrease its porosity can result in the loss of plants' ability to control the wind. Furthermore, in higher wind velocities even though both plants lose their frontal area simultaneously, with porosity, greater reduction in porosity and lower lose of projected area by *L. lucidum* compared to *C. bipinnatus* (Supplementary Fig. S7d) minimizes the chance of airflow and blown particles to pass through its canopy. Thus *L. lucidum* has greater ability to affect the sediment transport system.

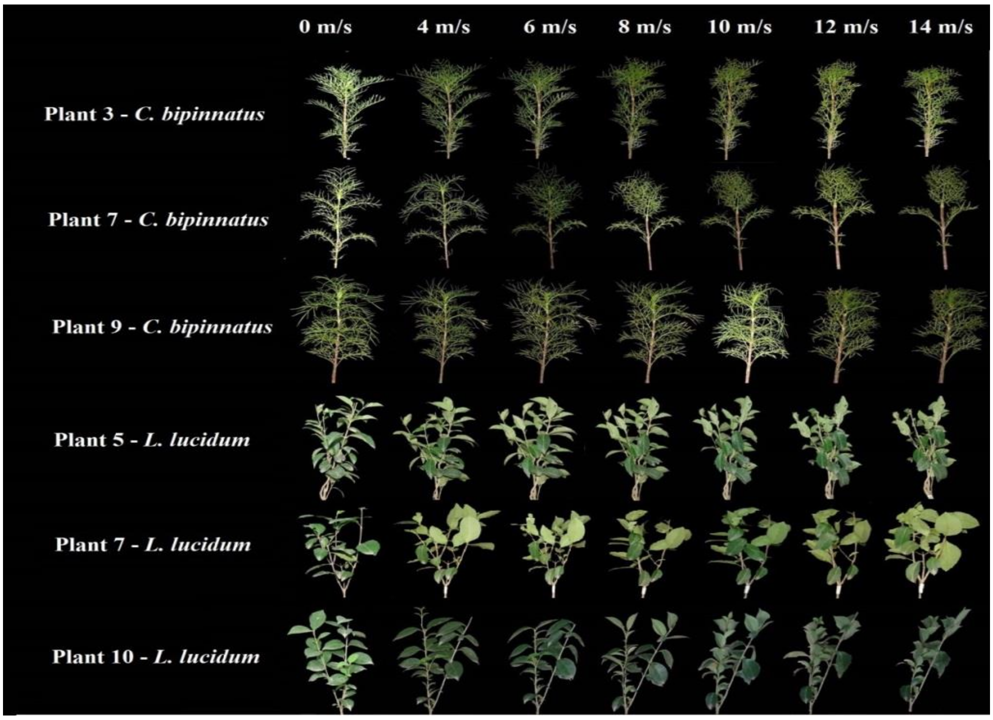

Supplementary Fig. S6 | Reconfiguration of plants. The response of plant numbers 3, 7 and 9 of *C. bipinnatus* and plant numbers 5, 7 and 10 of *L. lucidum* when subjected to free-stream velocities of $U_\delta = 0$, 4, 6, 8, 10, 12 and 14 ms^{-1}.

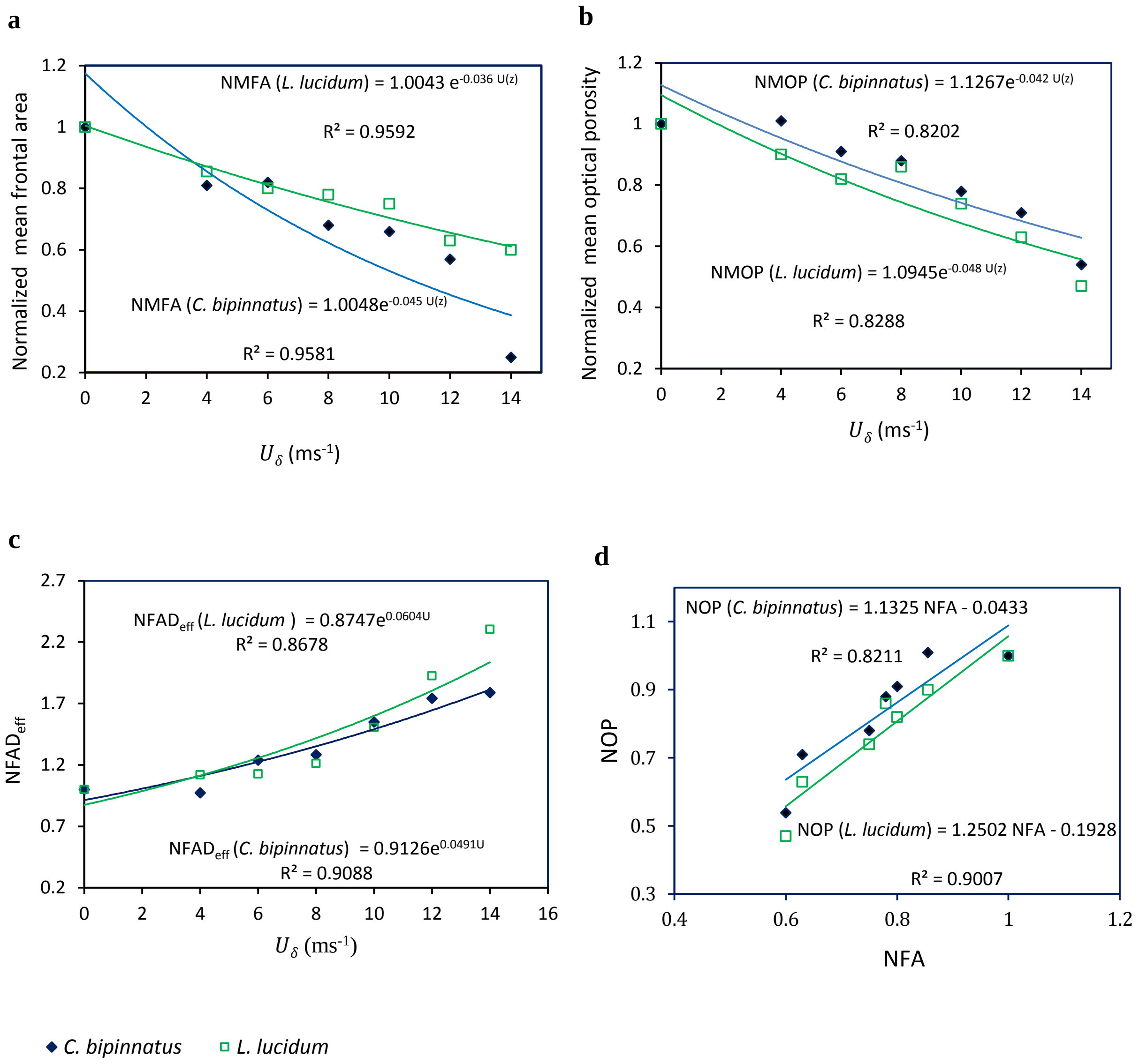

Supplementary Fig. S7. | **Aerodynamic responses of *C. bipinnatus* and *L. lucidum* to airflow.** (a,b) The exponential relationship between normalized value of mean frontal area (NMFA) and optical porosity (NMOP) with wind speed. (c) Exponential increase of normalized frontal area display ($NFAD_{eff}$) with increasing wind speed. (d) Simultaneous decrease of OP with FA with increasing wind speed.

5. Decreasing wind velocity with increasing plant density

The results show that by increasing the horizontal vegetation cover from $C_v \sim$ 10% to $C_v \sim$ 28% and $C_v \sim$ 56%, wind velocity in the canopies of both plant types decreases by about two or three times that of the equivalent unplanted condition (Supplementary Table S5 and Table S6). Changing the surface cover from an unplanted configuration ($C_v \sim$ 0%) to a high density configuration ($C_v \sim$56%) increases the roughness of the surface. This results in greater drag force being exerted on airflow by plants[22] within the canopies. Consequently, vegetation-cover density is a determining factor influencing different aspects of flow dynamics within the vegetative canopy[23,24] and especially in decreasing wind velocity. This wind velocity decrease with increasing surface coverage results in a changing drag coefficient and aerodynamic roughness length, and has been reported in previous studies[10,11,25-27].

Supplementary Table S5

Decrease in wind velocity (%) with horizontal vegetation coverage for *C. bipinnatus* in various wind velocities

**Wind velocity**	**Height**	**Decrease in wind velocity (%)**		
		$C_v \sim$ **10%**	$C_v \sim$ **28%**	$C_v \sim$ **56%**
$U_\delta = 6\ \mathrm{m\ s^{-1}}$	$z/h = 0.5$	23.51	35.97	52.47
	$z/h = 0.8$	37.62	44.79	59.57
$U_\delta = 8\ \mathrm{m\ s^{-1}}$	$z/h = 0.5$	6.92	43.14	49.80
	$z/h = 0.8$	37.62	44.79	59.57
$U_\delta = 12\ \mathrm{m\ s^{-1}}$	$z/h = 0.5$	9.22	51.18	52.48
	$z/h = 0.8$	24.16	52.09	57.95
$U_\delta = 14\ \mathrm{m\ s^{-1}}$	$z/h = 0.5$	21.76	47.6	53.74
	$z/h = 0.8$	28.09	59.48	62.02

Supplementary Table S6

Decrease in wind velocity (%) with horizontal vegetation coverage for *L. lucidum* in various wind velocities

**Wind velocity**	**Height**	**Decrease in wind velocity (%)**		
		$\boldsymbol{C_v}$ **~ 10%**	$\boldsymbol{C_v}$ **~ 28%**	$\boldsymbol{C_v}$ **~ 56%**
$\boldsymbol{U_\delta = 6\ \mathrm{m\ s^{-1}}}$	$z/h\ = 0.5$	25.85	41.4	54.4
	$z/h\ = 0.8$	39.01	60.38	67.64
$\boldsymbol{U_\delta = 8\ \mathrm{m\ s^{-1}}}$	$z/h\ = 0.5$	23.64	52.65	54.21
	$z/h\ = 0.8$	39.01	60.38	67.64
$\boldsymbol{U_\delta = 12\ \mathrm{m\ s^{-1}}}$	$z/h\ = 0.5$	21.02	54.13	56.41
	$z/h\ = 0.8$	43.04	63.02	63.89
$\boldsymbol{U_\delta = 14\ \mathrm{m\ s^{-1}}}$	$z/h\ = 0.5$	35.9	60.26	63.34
	$z/h\ = 0.8$	44.05	53.63	63.04

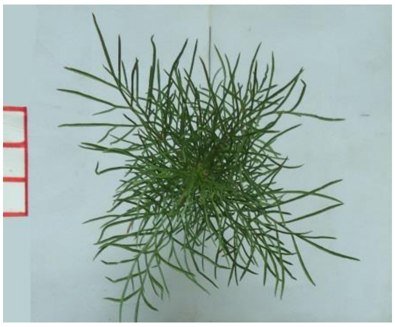

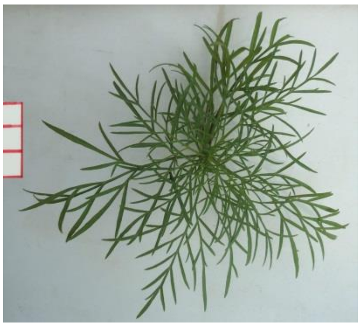

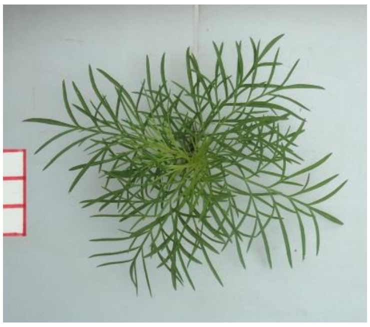

Plant 1 (ID 2) **Plant 2 (ID 9)** **Plant 3 (ID 16)**

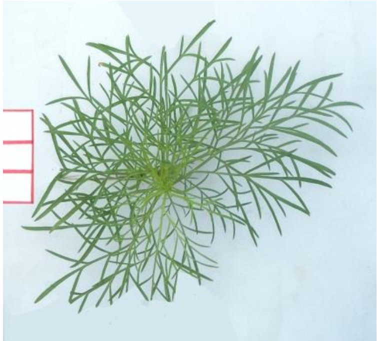

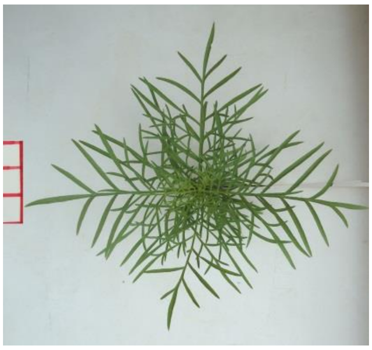

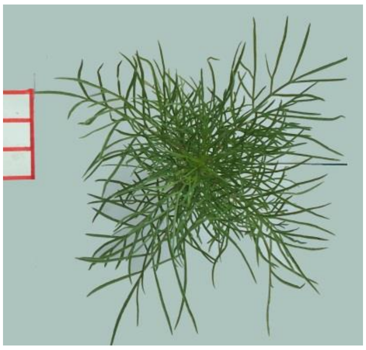

Plant 4 (ID 23) **Plant 5 (ID 30)** **Plant 6 (ID 37)**

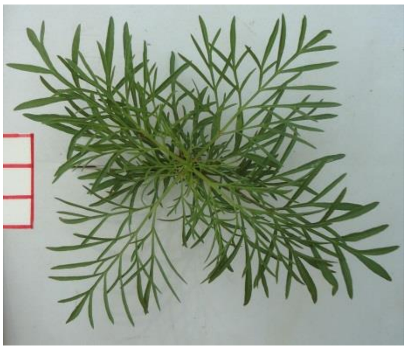

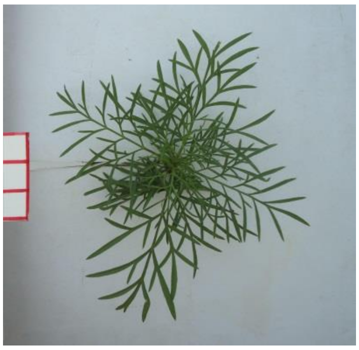

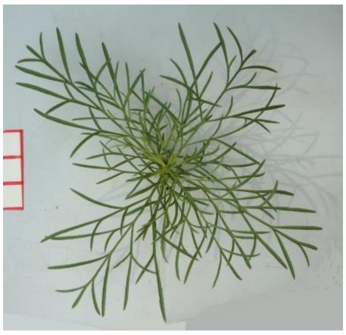

Plant 7 (ID 44) **Plant 8 (ID 51)** **Plant 9 (ID 58)**

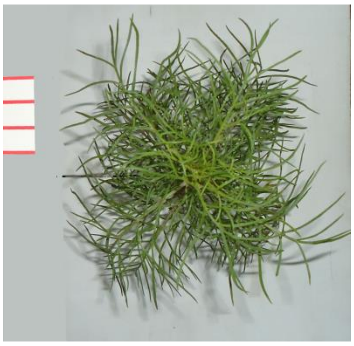

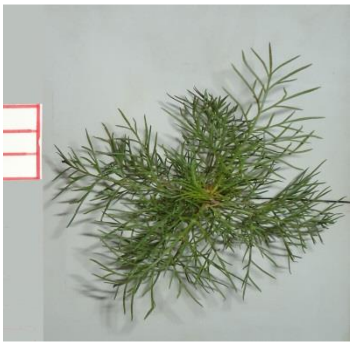

Plant 10 (ID 65) **Plant 11 (ID 72)**

Supplementary Fig. S8. Top view of the plants of *C. bipinnatus*. The eleven plants of *C. bipinnatus* which were selected to estimate mean canopy cover.

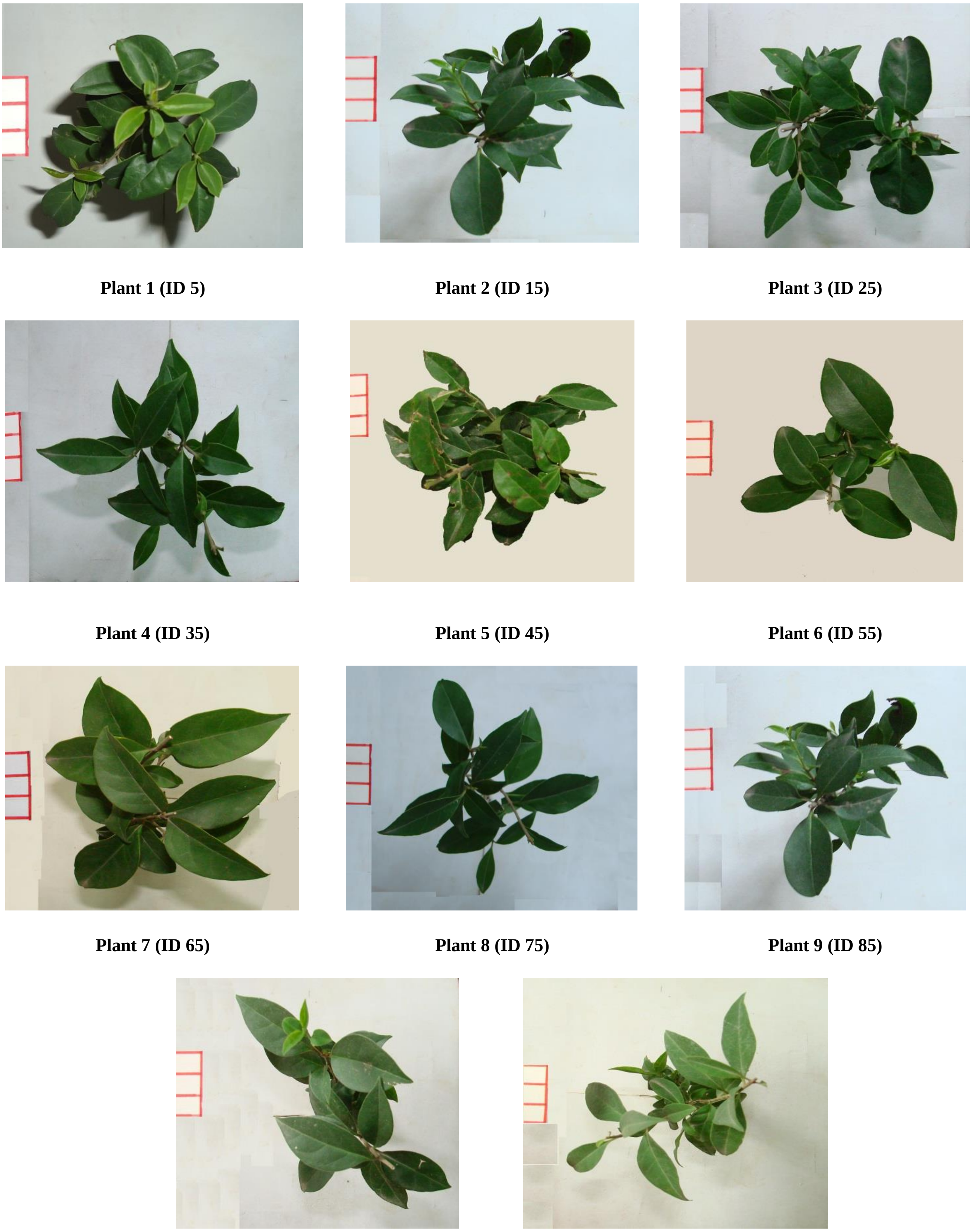

Plant 1 (ID 5) **Plant 2 (ID 15)** **Plant 3 (ID 25)**

Plant 4 (ID 35) **Plant 5 (ID 45)** **Plant 6 (ID 55)**

Plant 7 (ID 65) **Plant 8 (ID 75)** **Plant 9 (ID 85)**

Plant 10 (ID 95) **Plant 11 (ID 105)**

Supplementary Fig. S9. Top view of the plants of *L. lucidum*. The eleven plants of *L. lucidum* which were selected to estimate mean canopy cover**.**

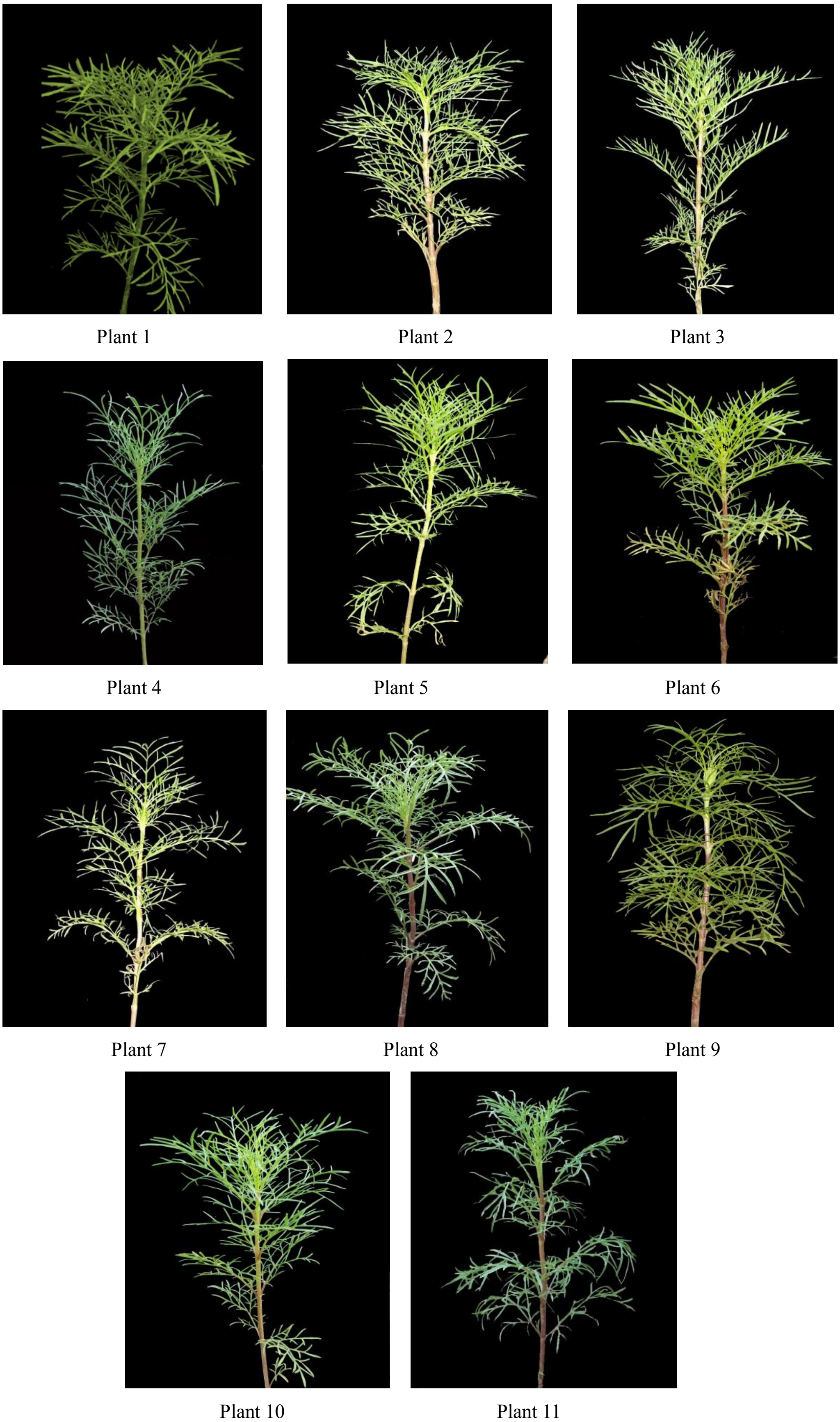

Supplementary Fig. S10. Frontal view of the plants of *C. bipinnatus*. The eleven plants of *C. bipinnatus* which were selected to estimate mean frontal area.

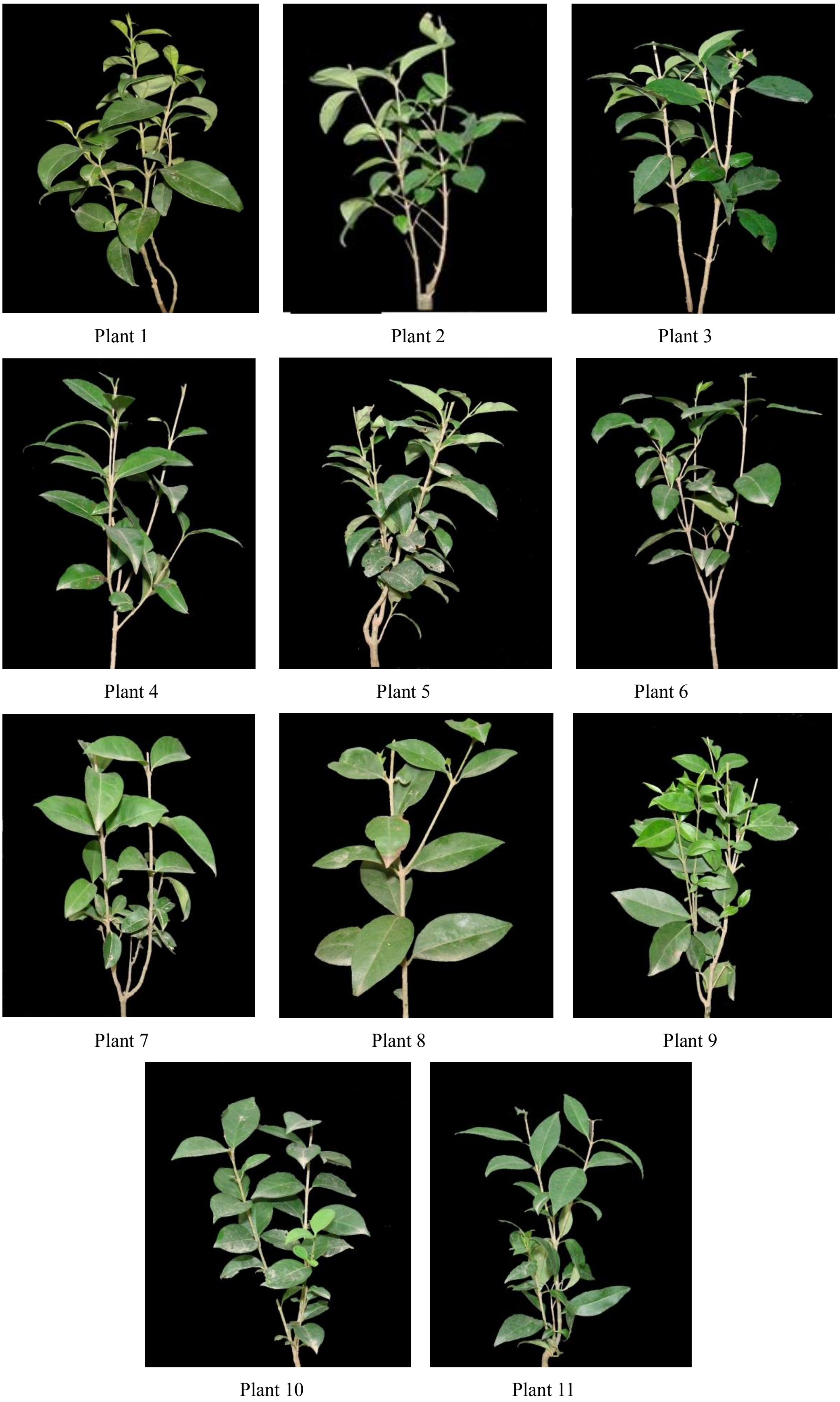

Supplementary Fig. S11. Frontal view of the plants of *L. lucidum*. The eleven plants of *L. lucidum* which were selected to estimate mean frontal area.

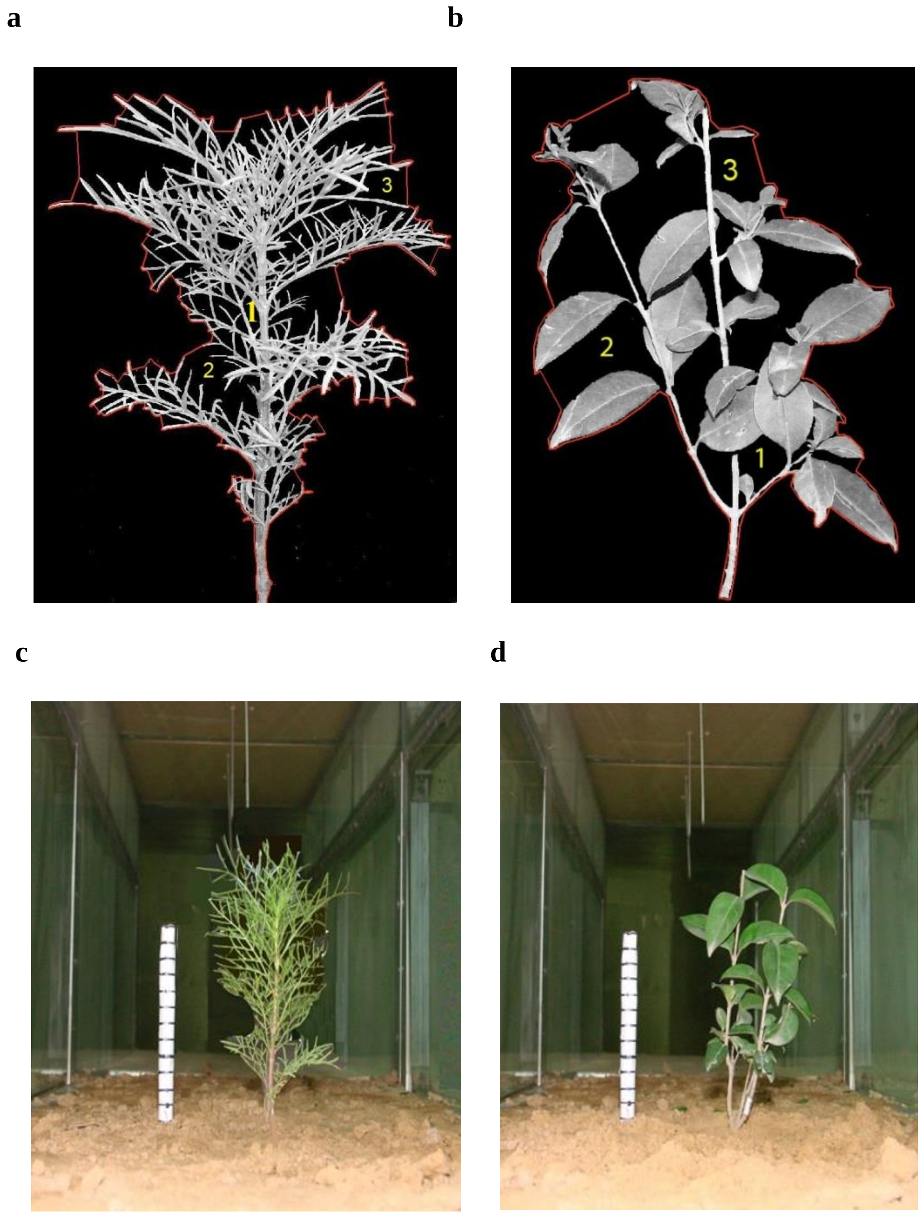

Supplementary Fig. S12. Measurement of three pore types. (a) *C. bipinnatus*. (b) *L. lucidum*. (c) *C. bipinnatus* subjected to the wind and (d) *L. lucidum* subjected to the wind in the wind tunnel.

Supplementary Table S7

Change of frontal areas (FA) of *C. bipinnatus* and *L. lucidum* when wind speed is increased over the range of $U_\delta = 0$ m s^{-1} to U_δ =14 m s^{-1}.

Plants	Loss of frontal area (%) when wind speed increases from $U_\delta = 4\ \mathrm{ms^{-1}}$ to $U_\delta = 14\ \mathrm{ms^{-1}}$	Frontal areas changes when wind speed increases from $U_\delta = 4\ \mathrm{ms^{-1}}$ to $U_\delta = 14\ \mathrm{ms^{-1}}$
C. bipinnatus	42	from 52 cm^2 to 30 cm^2
L. lucidum	30	from 52 cm^2 to 37 cm^2

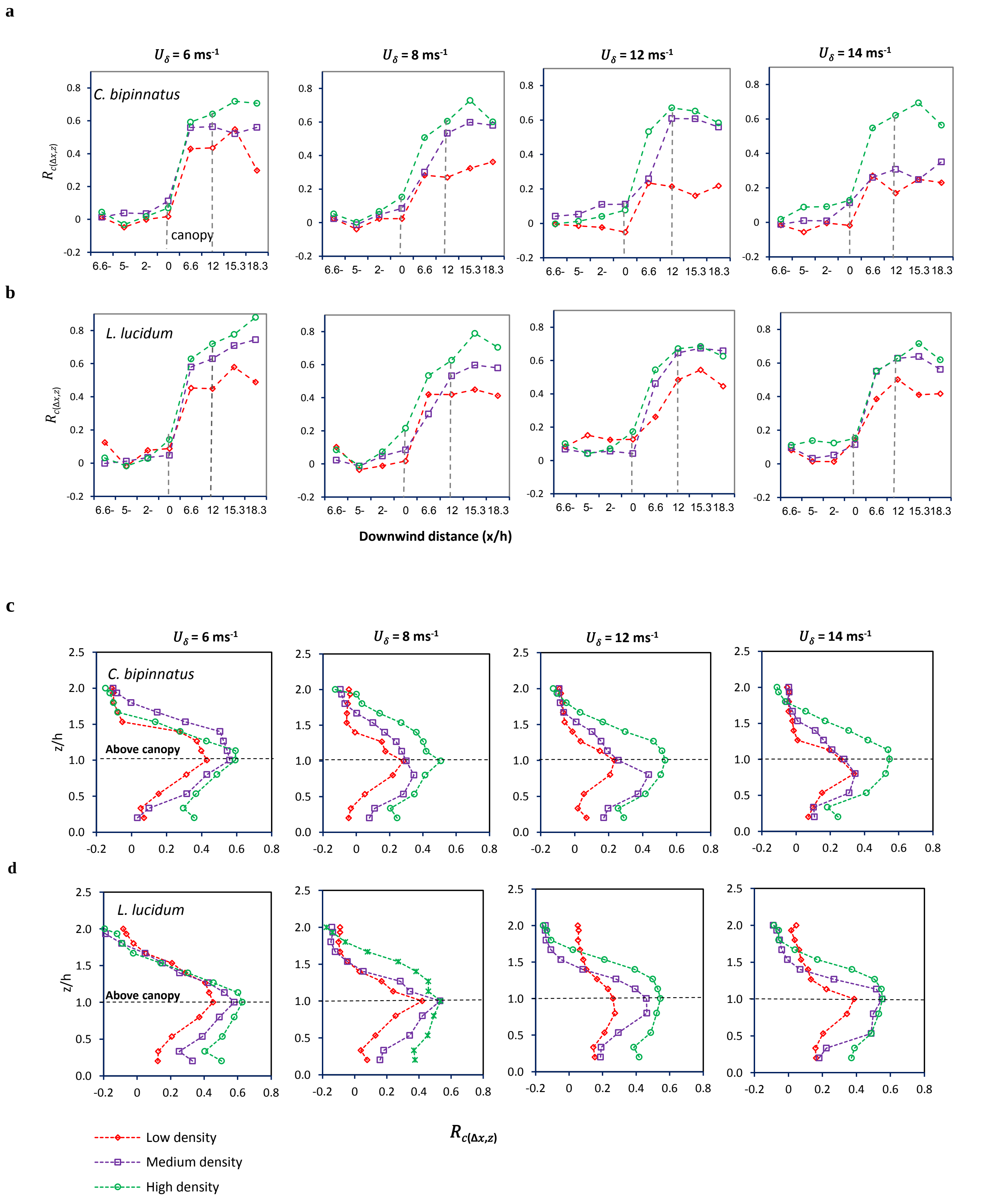

Supplementary Fig. S13 | Horizontal and vertical shelter effect of the plants as a function of λ in various wind speeds. (a) Horizontal shelter effect of *C. bipinnatus*. (b) Horizontal shelter effect of *L. lucidum*. (c) Vertical shelter effect of *C. bipinnatus*. (d) Vertical shelter effect of *L. lucidum*. The horizontal profiles are plotted at height of $z/h = 1$ and vertical profiles are plotted at downwind position of x/h = 6.6.

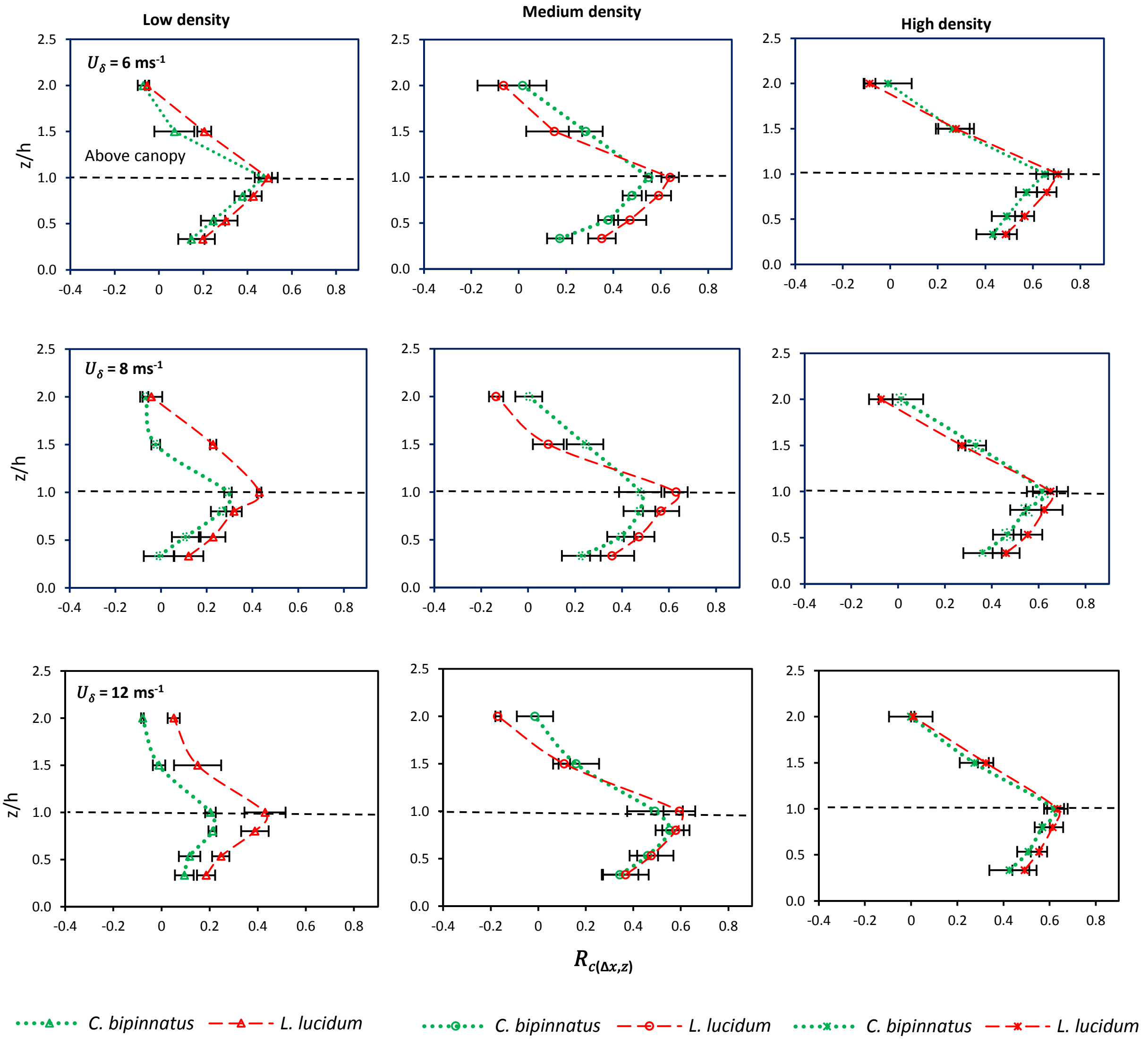

Supplementary Fig. S14 | Comparing the efficency of *C. bipinnatus* with *L. lucidum* in covering the surface. The vertical shelter effect is obtained by averaging the $R_{c(x,z)}$ values at the downwind positions of $x/h = 6.6 - 15.3$ in different densities and in wind velocities of $U_\delta = 6$, 8 and 12 ms^{-1}. Vertical bars show the calculated Standard Error of mean.

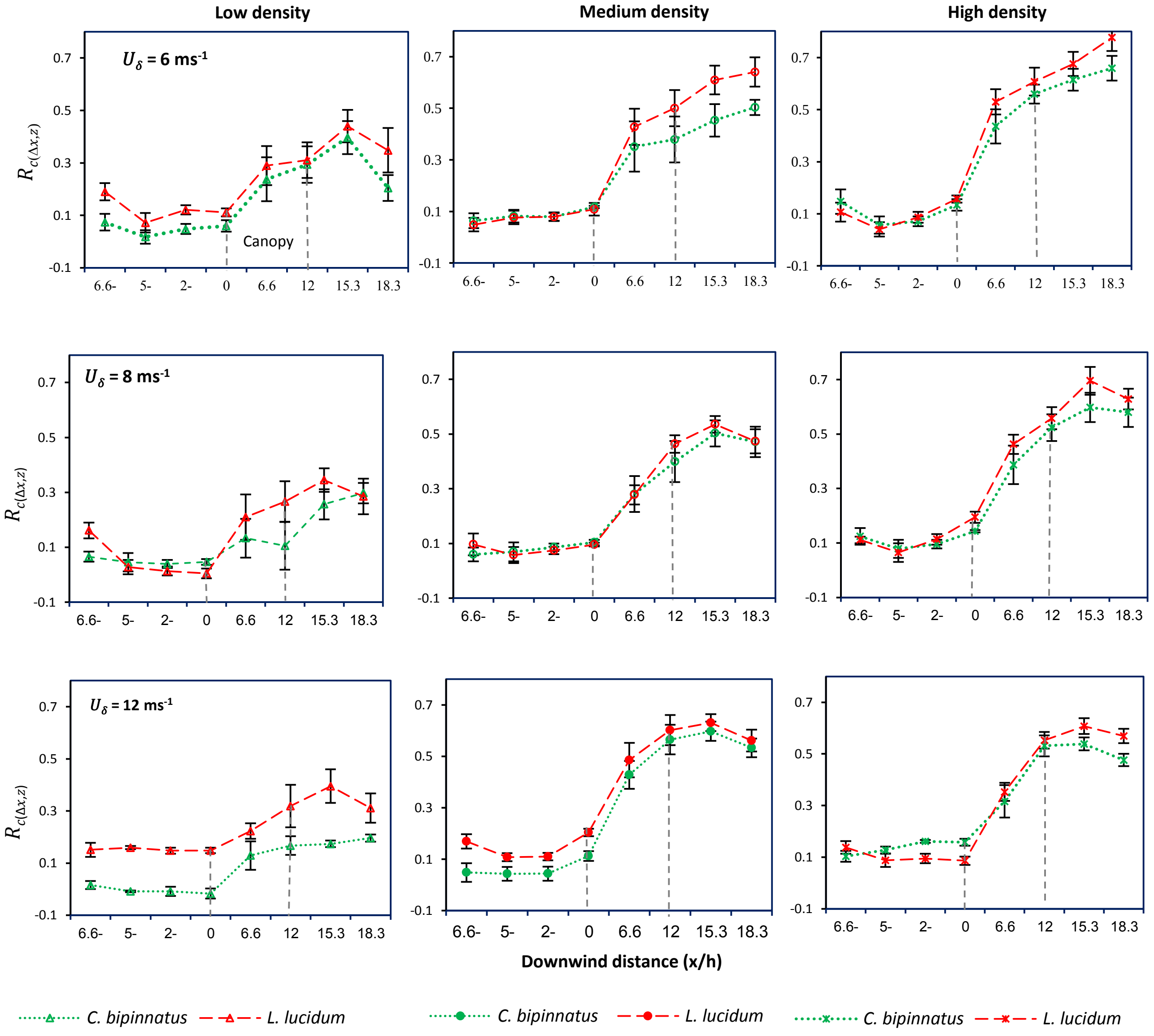

Supplementary Fig. S15 | Comparing the efficiency of *C. bipinnatus* with *L. lucidum* in covering the surface. The horizontal shelter effect is obtained by averaging the $R_{c(x,z)}$ values within a layer of $z/h = 0.3 - 1$ (below the canopy height) in different densities and in wind velocities of $U_\delta = 6$, 8 and 12 ms^{-1}. Horizontal bars show the calculated Standard Error of mean.

References

1 Brown, S., Nickling, W. & Gillies, J. A wind tunnel examination of shear stress partitioning for an assortment of surface roughness distributions. *J. Geophys. Res., Earth Surface*. **113**, (2008) F02S06, doi:10.1029/2007JF000790.

2 Webb, N. P., Okin, G. S. & Brown, S. The effect of roughness elements on wind erosion: The importance of surface shear stress distribution. *J. Geophys. Res. Atmos.* **119**, 6066-6084 (2014).

3 Marshall, J. K. Drag measurements in roughness arrays of varying density and distribution. *Agric. Meteoro.* **8**, 269-292 (1971).

4 Jia, Y., Sill, B. & Reinhold, T. Effects of surface roughness element spacing on boundary-layer velocity profile parameters. *J. Wind Eng. and Ind. Aerod.* **73**, 215-230 (1998).

5 King, J., Nickling, W. & Gillies, J. Aeolian shear stress ratio measurements within mesquite-dominated landscapes of the Chihuahuan Desert, New Mexico, USA. *Geomorphology* **82**, 229-244 (2006) doi:10.1016/j.geomorph.2006.05.004.

6 Udo, K. & Takewaka, S. Experimental study of blown sand in a vegetated area. *J. Coastal Res.* 1175-1182 (2007).

7 Sutton, S. & McKenna Neuman, C. Sediment entrainment to the lee of roughness elements: effects of vortical structures. *J. Geophys. Res.: Earth Surface* **113** (2008).

8 Burri, K., Gromke, C., Lehning, M. & Graf, F. Aeolian sediment transport over vegetation canopies: A wind tunnel study with live plants. *Aeolian Res.* **3**, 205-213 (2011).

9 Suter-Burri, K., Gromke, C., Leonard, K. C. & Graf, F. Spatial patterns of aeolian sediment deposition in vegetation canopies: Observations from wind tunnel experiments using colored sand. *Aeolian Res.* **8**, 65-73, doi:10.1016/j.aeolia.2012.11.002 (2013).

10 Walter, B., Gromke, C. & Lehning, M. Shear-stress partitioning in live plant canopies and modifications to Raupach's model. *Bound-Layer Meteor.* **144**, 217-241 (2012).

11 Walter, B., Gromke, C., Leonard, K. C., Manes, C. & Lehning, M. Spatio-temporal surface shear-stress variability in live plant canopies and cube arrays. *Bound-Layer Meteor.* **143**, 337-356 (2012).

12 Shirazi, R. & Shirazi, Z. Vegetation Dynamic of Southern Sistan during the Bronze Age: Anthracological Studies at Shahr-i Sokhta. *Iranian J. of Archae. Studies* **2**, 27-38 (2012).

13 Gross, G. A numerical study of the air flow within and around a single tree. *Bound-Layer Meteor.* **40**, 311-327 (1987).

14 Lancaster, N. & Baas, A. Influence of vegetation cover on sand transport by wind: field studies at Owens Lake, California. *Earth Surf. Process and Landf.* **23**, 69-82 (1998).

15 Righetti, M. Flow analysis in a channel with flexible vegetation using double-averaging method. *Acta Geophysica* **56**, 801-823 (2008).

16 Raupach, M., Gillette, D. & Leys, J. The effect of roughness elements on wind erosion threshold. *J. Geophys. Res. Atmos*. **98**, 3023-3029 (1993).

17 Wolfe, S. A. & Nickling, W. G. The protective role of sparse vegetation in wind erosion. *Prog. in Phys. Geogr.* **17**, 50-68 (1993).

18 Dubey, S. & Singh, V. K. Population dynamics of Aphis spiraecola Patch (Homoptera: Aphididae) on medicinal plant Cosmos bipinnatus in eastern Uttar Pradesh, India. *Adva. in Life Sci.* **1**, 54-58 (2011).

19 Leslie, P. Cosmos bipinnatus Mexican aster, garden cosmos. http://www.flowersociety.org/Cosmos-plant-study.htm (2005).

20 Vines, R. A. Trees of central Texas. University of Texas Press . (2010).

21 Nelson, G., Earle, C. J. & Spellenberg, R. *Trees of Eastern North America*. (Princeton University Press, 2014).

22 Crawley, D. & Nickling, W. Drag partition for regularly-arrayed rough surfaces. *Bound-Layer Meteor.* **107**, 445-468 (2003).

23 Raupach, M. R., Finnigan, J. & Brunet, Y. Coherent eddies and turbulence in vegetation canopies: the mixing-layer analogy. *Bound-Layer Meteor.* **78**, 351-382 (1996).

24 Finnigan, J. Turbulence in plant canopies. Annual review of fluid mechanics **32**, 519-571 (2000).

25 Raupach, M. R. Drag and drag partition on rough surfaces. *Bound-Layer Meteor.* **60**, 375-395 (1992).

26 Dong, Z., Gao, S. & Fryrear, D. W. Drag coefficients, roughness length and zero-plane displacement height as disturbed by artificial standing vegetation. *J. of Arid Environ.* **49**, 485-505 (2001).

27 Zhang, Q., Zeng, J. & Yao, T. Interaction of aerodynamic roughness length and windflow conditions and its parameterization over vegetation surface. *Chinese Sci. Bulletin* **57**, 1559-1567 (2012).
